# Activation in Right Dorsolateral Prefrontal Cortex Underlies Stuttering Anticipation

**DOI:** 10.1162/nol_a_00073

**Published:** 2022-08-17

**Authors:** Eric S. Jackson, Swethasri Dravida, Xian Zhang, J. Adam Noah, Vincent Gracco, Joy Hirsch

**Affiliations:** Department of Communicative Sciences and Disorders, New York University, New York, USA; Department of Psychiatry, Yale School of Medicine, New Haven, CT, USA; Haskins Laboratories, New Haven, CT, USA; McGill University, Montreal, Canada; Department of Neuroscience, Department of Comparative Medicine, Yale School of Medicine, New Haven, CT, USA; Department of Medical Physics and Biomedical Engineering, University College London, London, UK

**Keywords:** stuttering, anticipation, disfluency, fNIRS, frontoparietal network, error-likelihood monitoring, action-stopping

## Abstract

People who stutter learn to anticipate many of their overt stuttering events. Despite the critical role of anticipation, particularly how responses to anticipation shape stuttering behaviors, the neural bases associated with anticipation are unknown. We used a novel approach to identify anticipated and unanticipated words, which were produced by 22 adult stutterers in a delayed-response task while hemodynamic activity was measured using functional near infrared spectroscopy (fNIRS). Twenty-two control participants were included such that each individualized set of anticipated and unanticipated words was produced by one stutterer and one control participant. We conducted an analysis on the right dorsolateral prefrontal cortex (R-DLPFC) based on converging lines of evidence from the stuttering and cognitive control literatures. We also assessed connectivity between the R-DLPFC and right supramarginal gyrus (R-SMG), two key nodes of the frontoparietal network (FPN), to assess the role of cognitive control, and particularly error-likelihood monitoring, in stuttering anticipation. All analyses focused on the five-second anticipation phase preceding the go signal to produce speech. The results indicate that anticipated words are associated with elevated activation in the R-DLPFC, and that compared to non-stutterers, stutterers exhibit greater activity in the R-DLPFC, irrespective of anticipation. Further, anticipated words are associated with reduced connectivity between the R-DLPFC and R-SMG. These findings highlight the potential roles of the R-DLPFC and the greater FPN as a neural substrate of stuttering anticipation. The results also support previous accounts of error-likelihood monitoring and action-stopping in stuttering anticipation. Overall, this work offers numerous directions for future research with clinical implications for targeted neuromodulation.

## INTRODUCTION

Stuttering is a complex neurodevelopmental communication disorder that often negatively impacts social, emotional, and professional opportunities for more than 50 million adults worldwide. The disorder manifests itself to listeners as intermittent interruptions in speech production including part-syllable repetitions and audible and inaudible prolongations of sounds. However, these behaviors do not always accompany stuttering events because most, if not all, stutterers develop the remarkable ability to anticipate stuttering. As a result, they can alter their speech plan prior to execution by, for example, avoiding, stalling, or using a speaking strategy (e.g., pull-out, easy onset; Jackson et al., 2015, 2019). This potential discrepancy between the internal experience of the speaker (i.e., sensing upcoming speech breakdown) and how stuttering manifests itself to the listener (i.e., as something out of the ordinary) has important clinical implications because how individuals respond to anticipation shapes their communicative experiences. Behavioral and qualitative investigations of anticipation, especially in recent years, have improved our understanding of the phenomenon, but it is critical to augment this evidence with a neural account of stuttering anticipation, especially given the covert nature of anticipation. The purpose of this study was to initiate a brain-based understanding of stuttering anticipation by linking neural activation to self-reported anticipation and subsequent stuttering behaviors.

### The Anticipation of Stuttering

*Stuttering anticipation* refers to the sense or prescience that upcoming speech will be stuttered, should the speaker execute their speech plan as originally intended without alterations (Jackson et al., 2015; Wingate, 1975). Anticipation occurs on a temporal continuum from a longer-term or looming sense of impending stuttering to a shorter-term immediate sense of upcoming stuttering. For example, a speaker may anticipate a word months in advance of saying it (a student knowing at the beginning of the semester that they have to say a certain word in a presentation at the end of the semester); minutes or seconds before (when they are about to introduce themselves); or immediately before executing speech. Anticipation is driven by error-likelihood monitoring whereby the speaker learns associations between “errors” (i.e., stuttered utterances) and listener reactions or other environmental consequences, thereby learning to predict the occurrences of these errors (Arenas, 2012, 2017; Garcia-Barrera & Davidow, 2015). While adult stutterers, as a group, predict stuttering with high accuracy in experimental settings (greater than 90% accuracy; Knott et al., 1937; Milisen, 1938; Van Riper, 1936), there is a range in which speakers report anticipating stuttering, from, “sometimes” to “always” (Jackson et al., 2015). Anticipation is a relatively stable feature such that *anticipated* or *feared* words are stuttered in experiments even three months after they are identified by participants (Mersov et al., 2018). Arguably most important in the speaker’s experience is how they learn or choose to respond to anticipation, whether by avoiding, approaching, or implementing physical speaking strategies that prevent stuttering from coming to the surface (Jackson et al., 2015, 2019). In this way, responding to anticipation is mediated by cognitive control.

### Cognitive Control

Cognitive control refers to the ability to orchestrate brain functions to complete a given task or reach a certain goal (Miller, 2000). Cognitive control encompasses planning, initiating, and inhibiting actions or tasks, and being flexible and vigilant to tasks in response to environmental demands (Niendam et al., 2012). All of these processes are involved in responding to stuttering anticipation. For example, when a stutterer knows that they are going to stutter, they must initiate (or choose not to initiate) a response which may include avoidance or using a speaking strategy (Jackson et al., 2015, 2019); they may inhibit responses due to fear of negative reactions from the listener; they must be flexible with the challenge at hand (i.e., not being able to say what they want to say when they want to say it); and they must remain vigilant to their goal (i.e., producing speech). While numerous studies have examined cognitive control in children and adults who stutter (for review, see Anderson & Ofoe, 2019), no studies have assessed the relationship between cognitive control and anticipation directly.

### A Potential Neural Substrate of Stuttering Anticipation

While anticipation is pervasive in the stuttering experience and contributes significantly to the negative impact on quality of life for stutterers (Jackson et al., 2015; Tichenor & Yaruss, 2019), the neural underpinnings of anticipation and related cognitive control processes are unknown. Neurofunctional investigations of stuttering have instead focused on the speech motor network, revealing atypical activation in left perisylvian and motor areas along the arcuate and superior longitudinal fasciculus, and their homologous regions in the right hemisphere, as well as atypical activity in basal ganglia and cerebellum (Braun et al., 1997; S.-E. Chang et al., 2011; De Nil et al., 2000; Fox et al., 2000; Kell et al., 2009; Neef et al., 2016; Neumann et al., 2004; Preibisch et al., 2003; Toyomura et al., 2018). Some of these studies reported significant findings outside of the speech motor network in areas related to cognitive control, even though these areas were not the focus of those investigations. For example, it is widely known that the right dorsolateral prefrontal cortex (R-DLPFC) plays a critical role in cognitive control processes (Koechlin et al., 2003; MacDonald et al., 2000; Miller, 2000; Ridderinkhof et al., 2004). Stutterers exhibit elevated activation in the R-DLPFC (Kell et al., 2009; Lu et al., 2009; Neef et al., 2018), and treatment temporarily reduces activity in R-DLPFC while reducing stuttering symptoms (De Nil et al., 2004; Neumann et al., 2005). Kell et al. (2009) also found that stutterers who reported recovering from stuttering without treatment did not show elevated activation in R-DLPFC, suggesting that these patterns reflect compensatory efforts not learned in therapy (e.g., avoiding, stalling, or using other self-learned speaking strategies). However, because anticipation and the R-DLPFC were not the focus of these studies, the potential relevance of the R-DLPFC to stuttering anticipation, and to stuttering more broadly, is unknown.

We focus primarily on the R-DLPFC in this study, but other regions and networks are likely recruited during anticipation and responding to anticipation. Modern imaging and computational approaches, including task-induced and task-free connectivity analysis, have identified multiple non-overlapping and distributed brain networks that underlie cognitive control, including the frontoparietal network (FPN), salience network, cingulo-opercular network, and dorsal and ventral attention networks (D’Esposito & Postle, 2002; Menon & D’Esposito, 2021; Niendam et al., 2012). The FPN, which includes the R-DLPFC and right supramarginal gyrus (R-SMG) (Menon & D’Esposito, 2021), is particularly relevant because it co-activates with multiple other networks (e.g., salience network, cingulo-opercular network) to carry out the diverse processes associated with cognitive control (Marek & Dosenbach, 2018). The FPN initiates and flexibly modulates interactions between the salience and cingulo-opercular networks (Marek & Dosenbach, 2018), which both include the anterior cingulate cortex (ACC). ACC underlies error-likelihood monitoring (Brown & Braver, 2005): DLPFC co-activates with ACC, whereby ACC underlies the detection of errors in response to unintended outcomes and generates error signals, and DLPFC holds task-relevant information in working memory and initiates subsequent actions (Alexander & Brown, 2015; Holroyd & Yeung, 2012). It is reasonable to propose that the ACC underlies stuttering anticipation—the recognition of the breakdown or “glitch” in speech-language planning—and reasonable to predict that the R-DLPFC underlies initiating a response to this breakdown. Given the strong bidirectional connections within the FPN (Goldman-Rakic, 1988; Menon & D’Esposito, 2021; Mesulam, 1998), particularly between R-DLPFC and R-SMG, it is also reasonable to hypothesize that anticipation destabilizes these connections, resulting in altered connectivity.

While it was not possible in the current design to disentangle anticipation and responding to anticipation, the reader should be aware of these somewhat distinct but overlapping processes. We conceive of anticipation as an event—the point in time at which the speaker becomes aware that should they proceed as planned, they will overtly stutter—and responding to anticipation as the constellation of processes, involving the cognitive control system, that underlies how a speaker chooses to proceed in light of the knowledge that they are likely about to stutter. In the current design and given the hemodynamic lag associated with fNIRS, it was not possible to disentangle anticipation and responding to anticipation, and thus, the current study examined the processes involved in anticipation generally. Techniques with better temporal resolution than functional near-infrared spectroscopy (fNIRS; i.e., magnetoencephalography/electroencephalography (MEG/EEG)) are more suited to disentangle these two processes.

The current study examined the relationship between R-DLPFC and stuttering anticipation, which may clarify the significance of previous and seemingly incidental findings of elevated activation in R-DLPFC in stutterers (e.g., Kell et al., 2009; Neef et al., 2018). In a first visit, we used a clinical interview to determine individual-specific anticipated and unanticipated words (Jackson et al., 2020). In a second visit, which occurred between three and 10 days after the first visit, we used fNIRS to measure cortical activation immediately prior to participants producing the anticipated and unanticipated words in a delayed-response task. We focused on superficial cortical structures in part due to imaging depth restrictions associated with fNIRS. While we were not able to measure activation from deeper structures such as ACC, fNIRS offered several advantages compared to other techniques (e.g., functional magnetic resonance imaging (fMRI), EEG, MEG), including (1) robustness to speech movement artifact, and (2) allowing participants to produce speech while they sat upright and across from a communicative partner, which increased the likelihood of anticipation. fNIRS has also been validated as a tool to measure DLPFC activation associated with anticipation (Vassena et al., 2019). A matched control group was included to test whether stutterers recruit R-DLPFC differently than non-stutterers. Each anticipated and unanticipated word list was produced by a stutterer and a control speaker. We conducted a region of interest (ROI) analysis of R-DLPFC and hypothesized that (1) anticipation would be associated with greater activation in R-DLPFC, reflecting cognitive control processes associated with responding to anticipation, and (2) stutterers would exhibit greater activation than control speakers in R-DLPFC during this same time period. We also assessed functional connectivity to test whether anticipation was associated with reduced intrinsic connectivity within the FPN, specifically between R-DLPFC and R-SMG.

## MATERIALS AND METHODS

This study was approved by the Institutional Review Boards at New York University and Yale University. Consent was obtained for all participants in accordance with the Declaration of Helsinki.

### Participants

Twenty-seven adult stutterers were recruited through the first author’s clinical network, mass emails distributed by Friends: The National Association of Young People Who Stutter and the National Stuttering Association, and by word of mouth. After the fNIRS screening (see below), 22 stutterers (9 female; mean age = 31.9, *SD* = 9.1; three left-handed) and 22 control speakers (10 female; mean = 27.4, *SD* = 8.0; three left-handed) participated in the study. Control participants were recruited after the stuttering participants so that they could be matched for age, gender, and stimuli (see below). Male-to-female ratio was lower than what is typically observed in the stuttering literature (59% vs. ∼75–80%). All participants were between the ages of 18 and 50, reported that American English was their primary language (multilingual was acceptable as long as English was learned during early childhood [younger than 6 years of age]), and reported negative histories of neurological, speech-language, psychological, learning, and hearing impairment. Participant characteristics, including age, gender, treatment history, and extent score from the Stuttering Anticipation Scale (SAS; Jackson et al., 2018) are included in Table 1.

**Table 1.** Participant data

**ID**		**Age**	**Gender**	**Treatment history**	**%TS**	**SAS**
1		29	F	on and off in elementary school	43%	50
2		35	F	7 years	36%	100
3		23	M	2 weeks (intensive program)	0%	76
4		34	M	6 years	0%	79
5		26	F	3 years	87%	95
6		48	F	roughly 2 years	80%	87
7		21	M	8 years	27%	90
8		23	M	2.5 years	23%	77
9		29	M	9 months	45%	80
10		34	M	2 years	98%	80
11		37	F	off and on for years	23%	75
12		39	F	12–15 years	76%	86
13		23	F	6+ years	93%	89
14		47	F	none	30%	98
15		42	M	8 years	15%	90
16		18	M	10 years, on and off	70%	75
17		30	M	approx. 10 years	51%	76
18		29	M	about 7 years	25%	67
19		39	F	about 6 months	55%	76
20		25	M	about 7 years (on and off)	21%	70
21		22	M	none	55%	77
22		50	F	3 months	15%	99
	*M*	32			40%	81
	*SD*	9			30%	12

*Note*. Treatment history descriptions written as reported by participants. TS = Trials Stuttered; SAS = Stuttering Anticipation Scale (extent score out of 100, 0 = *never*, 100 = *always*). ID = participant; *M* = mean; *SD* = standard deviation.

#### First visit (stutterers only)

The first visit comprised (1) the stuttering assessment, (2) the clinical interview to determine participant-specific stimuli, and (3) the screening for fNIRS. Only the stuttering group participated in two visits; the fNIRS screening for the control group took place on the same day as fNIRS testing.

#### Diagnostic assessment

The stuttering group was identical to that in Jackson et al. (2020). That study validated the clinical interview used here, and the only overlapping data between that study and the current study are the speech classification data (stuttered/ambiguous/fluent, interrater reliability). Stuttering was diagnosed by the first author, an American Speech-Language-Hearing Association certified speech-language pathologist (SLP) with more than 10 years of expertise in stuttering intervention.

All stuttering participants (1) self-reported as a person who stutters; and (2) exhibited three or more stuttering-like disfluencies (Yairi & Ambrose, 1992) with temporally aligned physical concomitants (e.g., eye blinking, head movements) during a 5–10 min conversation. Participants also completed the SAS (Jackson et al., 2018), which provided self-report ratings of the extent of anticipation based on a 0–100 (*never–always*) visual analog scale (“How much do you anticipate stuttering?”).

#### Clinical interview

The clinical interview was described previously in Jackson et al. (2020), and will be described briefly here. Interviews were conducted at New York University and Yale University, and for two participants remotely. The purpose of the interview was to identify 10 words that the participants anticipated they would stutter (hence: *anticipated words*) and 10 words that they did not anticipate they would stutter (hence: *unanticipated words*), resulting in individual lists for each participant. This approach extends previous methods for identifying anticipated/unanticipated words (Bowers et al., 2012; Mersov et al., 2018; Wymbs et al., 2013), by including clinical inference (e.g., asking participants whether stuttered words/sounds not immediately identified by participants as anticipated or “feared” words/sounds should be included as anticipated words), as well as using counseling techniques to create an environment in which participants were comfortable to identify/reveal feared words. The words were used to create the stimuli for fNIRS testing, which consisted of short questions or sentence completions that would require the participant to produce the words. Anticipated and unanticipated words were matched for length (number of syllables). Each stutterer was matched with a control participant who produced the same set of words.

#### fNIRS screening

The goal of the fNIRS screening was to determine whether a reliable hemodynamic signal could be acquired from each participant. This is because factors such as bone density and skull thickness weaken fNIRS signals (Krall & Dawson-Hughes, 1993; Okada & Delpy, 2003). Screening fNIRS participants limits acquisition of invalid data (Zhang et al., 2017). The screening consisted of a finger tapping task during which participants were required to tap their fingers in an alternating pattern for 15 s then rest for 15 s, for a total of 3 min (right hand). Typically, this task elicits a robust response in left motor/premotor areas. Here, a response was determined to be reliable if there was sufficient separation between oxyhemoglobin (HbO) and deoxyhemoglobin (HbR) signals in any channel in left motor/premotor areas based on visual inspection of the event-triggered average. Importantly, potential participants were excluded before the study began. That is, no participants were excluded from the study after their signals were determined to be reliable. Twenty-seven stutterers were screened, and 22 participated in the study; twenty-eight controls were screened, and 22 participated in the study.

#### Second visit

The second visit (for the stuttering group) occurred between 3 and 10 days after the first visit, and included fNIRS testing in the Brain Function Laboratory at Yale. Participant-specific stimuli were created between the first and second visits based on the anticipated and unanticipated word lists established during the clinical interview (first visit). Each word list was used to create the stimuli for one stuttering and one control speaker. Stimuli included simple questions or sentence completions that required one-word responses (e.g., “You can fly in an ______” ➔ airplane. “What month comes after June?” ➔ July). All stimulus questions were approximately between 1 and 3 s. Participants were exposed to the stimuli before the experiment to minimize the potential impact of language retrieval and formulation processes (i.e., they became familiar with the questions and answers prior to the experiment).

**Figure 1.**
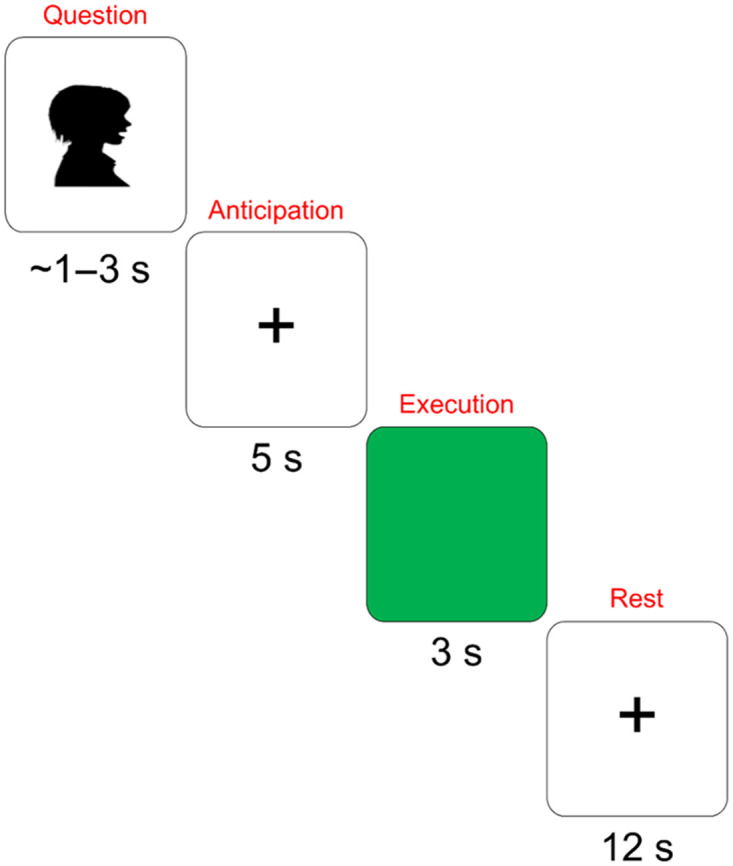
Task timeline. The question/sentence completion was presented auditorily. The anticipation period was 5 s; participants looked at a fixation cross during this time. The green screen signaled participants to produce the word. Participants again looked at the cross during the rest period.

Figure 1 depicts the task timeline. The question was presented auditorily while participants viewed a cross on the monitor. Verbal responses were delayed by 5 s. Participants responded when the screen turned green. Participants were asked to look straight ahead and to try to remain still. The paradigm included interactive and alone conditions, but the condition contrast was not the focus of the current study. The two conditions were pooled in all analyses. During the interactive condition, participants responded to questions asked by the examiner, who was seated directly across from the participant and in full view. Participants were instructed to look at the examiner while responding (e.g., “make sure you’re looking at me when you respond”). The experimenter was given a cue on a separate monitor (unseen by the participant) just before the go signal was presented, allowing him to look at the participants when they responded. During the alone condition, participants responded to prerecorded stimuli, the same questions as asked by the examiner as described above, while alone in the testing room. Questions during the interactive condition were matched, to the best of the examiner’s ability, to the prerecorded questions in terms of duration and prosody. The paradigm included a total of 80 trials: 4 interactive runs, 4 alone runs; each run included 10 words. Each word was produced 4 times.

### Data Acquisition

#### Behavioral

Speech data were acquired via acoustic and video recordings. Acoustic signals were recorded using a head-mounted microphone with a pop-screen filter set at the same fixed distance for each participant. Video was captured using a Logitech c920 HD 1080p video camera mounted on the participant’s monitor.

#### Neural

Data collection methods have been described previously (e.g., Hirsch et al., 2018, 2021), and are also described here. Hemodynamic signals were acquired using an 80-fiber continuous-wave fNIRS system (Shimadzu LABNIRS, Kyoto, Japan) with a temporal resolution of 27 ms. Forty emitters and forty detectors were arranged in a 134-channel layout covering bilateral frontal, temporal, parietal, and occipital lobes. Depending on the size of the participant’s head, caps with optode distances of either 2.75 cm or 3 cm were used. Three wavelengths of light (780, 805, and 830 nm) were delivered by each LABNIRS emitter. Absorption was converted to concentration changes for deoxyhemoglobin (HbR) and oxyhemoglobin (HbO) using the Beer-Lambert Law (Matcher et al., 1995). After the experiment, anatomical locations of optodes were determined based on standard head landmarks (inion, nasion, top center [Cz], and left and right tragi) using a Patriot 3D Digitizer (Polhemus, Colchester, VT) and linear transform techniques (Eggebrecht et al., 2012; Ferradal et al., 2014; Okamoto & Dan, 2005). Montreal Neurological Institute (MNI) coordinates for the channels were obtained using NIRS-SPM (Ye et al., 2009) in MATLAB (Mathworks, Natick, MA), and corresponding anatomical locations were determined for each channel. See Table S1 for group median coordinates, atlas-based probabilities, and anatomical regions for each channel; see Figure S1 for a visual representation. (Supporting Information can be found at https://doi.org/10.1162/nol_a_00073.) Channels were clustered into anatomical regions based on shared anatomy. The average number of channels per region was 2.69 ± 1.40.

### Data Processing

#### Behavioral

Errors, which comprised non-productions or incorrect productions due to participants forgetting answers or producing erroneous speech, were not included in any analyses (Table 2). Reaction time was calculated as the time between the go signal (i.e., the green screen) and speech onset as defined by the first articulatory movement or accessory behavior (Table 3). Articulatory onset was marked as the first articulatory movement based on visual inspection using Davinci Resolve (Black Magic Design, Australia), which allowed for frame-by-frame scanning (29.97 frames per second) of the recordings of participants’ faces. Interrater reliability between the first author and a SLP blind to the study yielded a Cohen’s weighted kappa of 0.89 (*p* < 0.05), indicating strong agreement (McHugh, 2012). We used articulatory onset because (1) inaudible sound prolongations (blocks) typically included observable movement such as posturing; and (2) it appeared that for some participants, video and audio were not synchronized due to technical error. Although determining neural correlates of stuttered speech was not the primary goal of this study, we include a comparison of stuttered and fluent speech for completeness. A three-point rating system was used to classify stuttering response type: 0 indicated *unambiguous fluency*; 1 indicated *ambiguity* (unclear whether stuttered or fluent); and 2 indicated *unambiguous stuttering* (Jackson et al., 2020).

#### Neural

Data processing was similar to that previously reported (Hirsch et al., 2017, 2018; Zhang et al., 2017). Baseline drift was removed using a NIRS-SPM detrending procedure. Global components were removed using a principal component analysis spatial filter (Zhang et al., 2016), which is comparable to using short-source channels (Noah et al., 2021). Channels were rejected if the root mean square (*RMS*) of the raw signal was 10 times greater than the group mean *RMS*, which resulted in a mean of 1.48 of 134 (1.1%) channels per participant rejected. Little to no motion artifact was observed in the data, based on visual inspection, likely because participants were instructed to remain still during the task. This is typical for compliant adult participants (Noah et al., 2021). Further, there was no speech movement observed during the anticipation phase. HbR and HbO signals were acquired. The fNIRS data sets for each subject were reshaped into 3D volume images for the general linear model (GLM) analysis using SPM8 (Wellcome Trust, London, UK). Beta value coordinates were converted to standard MNI space using NIRS-SPM (Ye et al., 2009). Contrast images were rendered on a standardized MNI brain template using a *p*-value threshold of 0.05 and cluster size threshold of 50 voxels, for visual representation. Anatomical locations of peak voxel activity were identified using the Brodmann area Talairach atlas (Lancaster et al., 2000).

### Statistical Analysis

#### Behavioral

To examine reaction time and stuttering response type (stuttered/ambiguous/fluent), linear mixed effects models were fit using the lme4 (Bates et al., 2014) and lmerTest packages in R (Kuznetsova et al., 2017; R Core Team, 2014). The MuMIn package (Barton, 2020) was used to calculate estimated R^2^ for model fit. For reaction time, the model included word type (anticipated/unanticipated), word length (number of syllables), trial, and stuttering response type as fixed factors, and participant as a random factor to account for expected variation due to individual differences. Wilcoxon rank sum tests were also used to assess reaction time differences for stuttered, ambiguous, and fluent speech. For stuttering response type, the model included word type, word length, and trial as fixed factors, and participant as a random factor.

#### Neural

All fNIRS analyses followed standard voxel-wise GLM techniques (Friston et al., 1994, 1995) adapted for fNIRS (e.g., Descorbeth et al., 2020; Hirsch et al., 2021). Analyses targeted the anticipation phase, i.e., the 5 s time window between the end of the question and the go cue (see Figure 1). All included voxels were within 2 cm from the cortical surface. The primary ROI analyses focused on the R-DLPFC; secondary ROI analyses examined activation in the right inferior frontal gyrus (R-IFG) and right pre-supplementary motor area (R-preSMA). Connectivity between the R-DLPFC and R-SMG was also assessed.

##### ROI analyses.

The mask for R-DLPFC was created by generating a 10 mm sphere using the MarsBar toolbox (Brett et al., 2002) and *xyz* coordinates [50 26 38] from Kell et al. (2009). Five ROI analyses of R-DLPFC were conducted.

(1) *Anticipated vs. Unanticipated*: Activation associated with anticipated vs. unanticipated words was compared by using SPM8 to convolve a 5 s block regressor during the anticipation phase, with a standard hemodynamic response function (HRF) that was fitted to the data. The first-level analysis yielded two beta values for each participant in each run (i.e., 10 trials per run), for anticipated and unanticipated words (five anticipated, five unanticipated). The results of the second-level contrast of anticipated vs. unanticipated words were projected onto a standardized MNI template image using SPM (*p* < 0.05 and cluster size of >50 voxels, uncorrected). Note that the “whole-brain” image was used only to test the ROIs; however, whole-brain results for this analysis, and all analyses below, are included as supplementary material. Significance for the ROI analysis was tested using a one-tailed *t* test in SPM (*p* < 0.05) by determining overlap between the second-level image (anticipated vs. unanticipated) and the mask. Two additional analyses were conducted to test whether the R-DLPFC ROI was significantly activated during the anticipation phase for anticipated and unanticipated words (separately). The second-level results, which compared activation for both anticipated and unanticipated words to rest, were projected onto the same MNI template, and the ROI analyses were carried out as described above.

(2) *Stuttered vs. Fluent*: To compare activation related to response type within stutterers (stuttered, ambiguous, fluent), the regressor was modulated in height (0 = *fluent*, 1 = *ambiguous*, 2 = *stuttered*) in the first-level analysis. The assumption was that stuttered speech yielded more (or less) activity than ambiguous responses, which yielded more (or less) activity than fluent responses, effectively providing a “contrast” of stuttered and fluent speech. Beta values were compared to zero using a one-tailed *t* test in SPM. The ROI analysis was conducted as described above.

(3) *Stutterers vs. Controls*: The ROI group level comparison was similar to the anticipated vs. unanticipated contrast described above, except for the second-level contrast. The 5 s block regressor was convolved with the HRF, irrespective of whether the word was anticipated. Results of the second-level contrast for stutterers vs. controls were projected onto a standardized MNI template image, and the ROI analysis was carried out as described above.

(4) *Task vs. Rest (controls only)*: We also conducted an analysis of the controls only, during the same 5 s window. The purpose of this analysis was to determine whether the R-DLPFC ROI was activated prior to speech execution, which could indicate whether R-DLPFC is involved in speech motor planning in unimpaired speakers. The second-level results, which compared activation of the controls to rest, were projected onto the same MNI template, and the ROI analysis was carried out as described above.

(5) *Interactive vs. Alone*: Finally, we compared activation between the interactive and alone conditions to justify our decision to exclude “condition” as a factor from all of the models. Confirmation of the null hypothesis would suggest that condition is not contributing to the anticipated and unanticipated responses.

We completed two additional ROI analyses. First, we used two control ROIs to confirm that our findings in R-DLPFC were specific to stuttering anticipation, and not, for example, general to stuttering or due to systemic artifact. These included L-DLPFC, the homologue of the R-DLPFC ROI, and right precentral gyrus (R-preCG) from Belyk et al. (2017), the most recent activation-likelihood meta-analysis of state stuttering (i.e., stuttered vs. fluent). ROIs were 10 mm spheres with centroids [−50 26 38] for L-DLPFC and [54 −14 34] for R-preCG. Second, we tested two key superficial cortical nodes of the action-stopping network (i.e., R-IFG and R-preSMA), as it has been proposed that action-stopping is associated with stuttering anticipation (Arenas, 2017; Hannah & Aron, 2021; Neef et al., 2018). Centroid coordinates were obtained from recent meta-analyses of stuttering: [46 23 −5] for R-IFG, included as a “state” finding in Belyk et al. (2015); and [15 13 59] for R-preSMA, included as a “trait” finding in Budde et al. (2014). Trait findings were used for R-preSMA because state findings were not reported in either meta-analysis. The coordinates were used to create 10 mm ROI spheres.

##### Functional connectivity.

Psychophysiological Interaction (PPI) analysis (Friston et al., 1994, 2003) was used to examine functional connectivity between R-DLPFC and R-SMG. PPI computations were performed on residual components of the modeled task (anticipated vs. unanticipated), after the GLM was effectively removed. Significant correlations are thought to reflect dynamic neural coupling, though not necessarily related to the task. PPI analysis was conducted using the gPPI toolbox (McLaren et al., 2012) with SPM8. The PPI analysis can be described by the following equations:

$$Y_{k}=H\left(x_{a}\right),$$ (1)

$$Y_{i}=\left[H\left(x_{a}*g_{p}\right)\right]*\beta_{i}+\left[H\left(g_{p}\right)\right]*\beta_{p}+Y_{k}*\beta_{k}+e_{i},$$ (2)

in which the HRF is represented by *H*, and *H*(*x*) is the convolution of signal *X* with kernel *H*. The demeaned time course is represented by *g*_*p*_ where 1 is task time and −1 is rest. β_*i*_ is the PPI beta value, whereas β_*p*_ is the beta value for the task, β_*k*_ is the beta value of the time course of the seed, and *Y*_*k*_ represents the fNIRS data collected at the seed region. Here, *k* is the functionally defined cluster for the seed region based on the GLM. *x*_*a*_ represents the estimated neural activity for the seed region; the residual error is represented by *e*_*i*_. R-DLPFC had ROI coordinates [50 26 38], and R-SMG coordinates were determined using the atlas from NIRS-SPM (Rorden & Brett, 2000) and creating a 10 mm sphere that was projected onto the brain surface. Each area was used both as a seed and a target, resulting in two comparisons in total. Two-tailed *t* tests were used to compare residual activity for anticipated versus unanticipated words, and the Holm-Bonferroni method was used to correct for familywise error rates.

## RESULTS

### Behavioral

Two linear mixed effects models (not included in Jackson et al., 2020) were fit to examine reaction time and response type. Mean reaction time across all stuttering participants was 348.27 ms (*SD* = 132.34 ms). See Table 2. Reaction time was significantly impacted by word type (anticipated/unanticipated), such that anticipated words had longer reaction times ($\hat{\beta }$ = 11.45, *t* = 2.13, *p* < 0.05). Reaction time was also impacted by trial ($\hat{\beta }$ = −0.34, *t* = −2.91, *p* < 0.01), such that reaction time decreased as the experiment progressed. Reaction time was not impacted by word length (syllables) ($\hat{\beta }$ = −0.07, *t* = −0.73, *p* > 0.05) or stuttering response type ($\hat{\beta }$ = −5.45, *t* = −1.54, *p* > 0.05), indicating that reaction time for stuttered trials was not significantly longer than that for fluent trials. *R*^2^ for the reaction time model was 0.24. In addition, post hoc Wilcoxon rank sum tests did not reveal differences in reaction time between stuttered and ambiguous trials (W = 203, *p* > 1.10), ambiguous and fluent trials (W = 190, *p* > 0.10), or stuttered and fluent trials (W = 152, *p* > 0.10). Interrater reliability for reaction time between the first author and a SLP blind to the study yielded a Cohen’s weighted kappa of 0.79 (*p* < 0.05), indicating moderate to strong agreement (McHugh, 2012).

**Table 2.** Reaction time means and standard deviations, as determined by first movement

**ID**	**Fluent**	***SD***	**Ambiguous**	***SD***	**Stuttered**	***SD***
1	352.14	103.71	286.03	69.08	297.39	108.48
2	362.68	115.64	307.75	101.19	368.22	97.08
3	398.99	126.61	300.33	168.84	–	–
4	311.76	120.47	467.18	47.19	–	–
5	–	–	–	–	–	–
6	386.14	90.07	517.24	50.61	458.53	109.47
7	341.00	187.38	406.51	132.49	272.23	87.77
8	310.22	72.77	296.29	60.60	300.33	62.43
9	370.68	109.21	433.81	129.24	324.43	104.50
10	–	–	–	–	282.50	81.94
11	302.33	75.50	361.51	114.57	367.07	144.78
12	333.70	100.11	305.89	64.95	262.28	94.69
13	360.40	148.11	300.33	75.91	303.49	69.47
14	480.53	147.94	524.39	195.26	560.34	154.65
15	327.57	115.33	322.58	70.79	294.77	111.89
16	290.32	99.55	340.85	68.24	282.73	69.12
17	281.56	87.84	302.18	84.48	330.44	140.16
18	421.30	132.68	446.17	208.43	361.51	134.58
19	515.72	122.92	517.24	306.75	489.97	134.20
20	323.34	76.61	260.29	27.92	288.55	110.64
21	304.64	160.21	133.48	47.19	285.78	97.98
22	362.01	125.17	310.76	147.09	367.07	95.85
–	358.90	–	352.49	–	336.10	–

*Note*. Data not obtained in empty cells due to technical complications. ID = participant; *SD* = standard deviation.

Table 3 shows the amount of stuttered, ambiguous, and fluent trials for each participant. For all trials, 43.6% were stuttered (2), 43.3% were fluent (0), and 13.1% were ambiguous (1), or not unambiguously stuttered or fluent (reported in Jackson et al., 2019; see Table 3). Interrater reliability between the first author and an SLP with 8 years of experience (blind to the study) yielded a Cohen’s weighted kappa of 0.85 (*p* < 0.05), indicating high agreement. The remaining response type data were not reported in Jackson et al. (2020). 53.9% of anticipated words were unambiguously stuttered and 33.4% of unanticipated words were unambiguously stuttered. 35.4% of anticipated words were unambiguously fluent whereas 51.2% of unanticipated words were unambiguously fluent. In addition, 10.7% of anticipated words were ambiguous and 15.41% of unanticipated words were ambiguous. It is important to note that words characterized as ambiguous using the Jackson et al. (2020) approach would most likely have been categorized as fluent with a standard binary stuttered/fluent distinction that is most commonly applied clinically. There was more stuttering for anticipated than unanticipated words, as expected ($\hat{\beta }$ = −0.36, *t* = −9.76, *p* < 0.001). There was also more stuttering for longer than shorter words ($\hat{\beta }$ = 0.11, *t* = 5.84, *p* < 0.001) and for earlier than later trials ($\hat{\beta }$ = −0.004, *t* = −4.42, *p* < 0.001), reflecting a reduction in stuttering over the course of the experiment. *R*^2^ for the stuttering response type model was 0.38. Pearson’s correlation test indicated that stuttering rate, expressed as the percentage of trials stuttered for each participant, and extent of anticipation for each participant (i.e., SAS extent score) were not related (*t* = 0.47, *p* > 0.10).

**Table 3.** Stuttering response type by participant, including percentages

**ID**	**Trials**	**Errors**	**Unambiguously fluent (0)**	**%**	**Ambiguous (1)**	**%**	**Unambiguously stuttered (2)**	**%**	**Anticipated words that were unambiguously stuttered**	**%**	**Unanticipated words that were unambiguously stuttered**	**%**	**Anticipated words that were unambiguously fluent**	**%**	**Unanticipated words that were unambiguously fluent**	**%**	**Anticipated words that were ambiguous**	**%**	**Unanticipated words that were ambiguous**	**%**
1	80	1	38	49%	7	9%	34	43%	26	65%	8	20%	13	33%	25	65%	1	3%	6	15%
2	80	4	38	53%	9	11%	29	36%	25	63%	4	10%	10	30%	28	75%	3	8%	6	15%
3	80	4	70	93%	6	8%	0	0%	0	0%	0	0%	38	98%	32	88%	1	3%	5	13%
4	80	3	75	98%	2	3%	0	0%	0	0%	0	0%	38	98%	37	98%	1	3%	1	3%
5	60	1	2	4%	5	6%	52	87%	29	97%	23	77%	0	3%	2	7%	0	0%	5	17%
6	70	3	7	10%	6	8%	54	80%	30	89%	24	71%	1	3%	6	20%	3	9%	3	9%
7	70	1	38	49%	12	15%	19	27%	17	49%	2	6%	11	34%	27	77%	6	17%	6	17%
8	80	1	27	35%	34	43%	18	23%	11	28%	7	18%	13	35%	14	35%	15	38%	19	48%
9	80	0	37	46%	7	9%	36	45%	24	60%	12	30%	13	33%	24	60%	3	8%	4	10%
10	80	2	0	3%	0	0%	78	98%	40	100%	38	95%	0	0%	0	5%	0	0%	0	0%
11	80	0	50	63%	12	15%	18	23%	7	18%	11	28%	27	68%	23	58%	6	15%	6	15%
12	80	1	7	9%	12	15%	60	76%	30	78%	30	75%	0	0%	7	18%	9	23%	3	8%
13	80	0	5	6%	1	1%	74	93%	38	95%	36	90%	2	5%	3	8%	0	0%	1	3%
14	80	1	47	60%	8	10%	24	30%	20	50%	4	10%	16	40%	31	80%	4	10%	4	10%
15	80	9	49	73%	10	13%	12	15%	11	28%	1	3%	20	58%	29	88%	6	15%	4	10%
16	80	0	10	13%	14	18%	56	70%	34	85%	22	55%	2	5%	8	20%	4	10%	10	25%
17	80	0	18	23%	21	26%	41	51%	24	60%	17	43%	8	20%	10	25%	8	20%	13	33%
18	80	1	30	39%	29	36%	20	25%	11	28%	9	23%	15	38%	15	40%	14	35%	15	38%
19	80	4	33	43%	2	3%	41	55%	33	90%	8	20%	3	10%	30	75%	0	0%	2	5%
20	80	0	58	73%	5	6%	17	21%	10	25%	7	18%	28	70%	30	75%	2	5%	3	8%
21	80	5	33	41%	2	4%	40	55%	24	68%	16	43%	13	33%	20	50%	0	0%	2	8%
22	60	2	33	43%	16	21%	9	15%	6	20%	3	10%	19	63%	14	50%	5	17%	11	40%
			736		222		742		458		284		301		435		91		131	
% across data set			43.30%		13.10%		43.60%		53.88%		33.41%		35.41%		51.18%		10.71%		15.41%	

*Note*. ID = participant.

### Neural

#### ROI analyses

**Figure 2.**
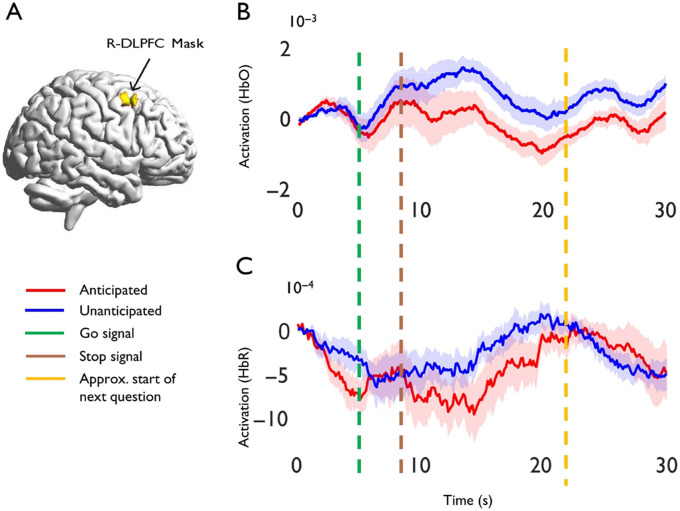
Region of interest analyses. (A) ROI mask for right dorsolateral prefrontal cortex (R-DLPFC) [58 26 38]. A 10 mm sphere was created using the MarsBar toolbox (Brett et al., 2002) and the xjView toolbox (https://www.alivelearn.net/xjview). The image was then projected onto the template using BrainNet Viewer (Xia et al., 2013). (B–C) Event-related averages of activity for the stuttering group within the R-DLPFC ROI for anticipated vs. unanticipated words, for Oxyhemoglobin (HbO) and DeOxyhemoglobin (HbR), respectively. 0 seconds is the beginning of the anticipation phase, and 30 s is shown because the hemodynamic response could feasibly last this long. HbR signals prior to sign reversal such that decreasing values reflect increases in activation strength. Shading represents standard error of the mean.

(1) *Anticipated vs. Unanticipated*: The primary contrast compared activation in the R-DLPFC mask associated with anticipated vs. unanticipated words. Anticipated words were associated with greater activation than unanticipated words (*p* = 0.0217) based on HbR, but not HbO (*p* > 0.05). Figure 2 includes the ROI illustration (A) and event-related averages for anticipated vs. unanticipated words, for HbO (B) and HbR (C), respectively. The HbR difference between event-related averages at 5 s (i.e., the end of the anticipation phase) for anticipated vs. unanticipated words approached significance (*t* = 1.67, *p* = 0.0567), though for HbO, signals were similar (*t* = 0.57, *p* > 0.05). Note that the remaining analyses in this section focus on HbR due to the null HbO results, as well as evidence suggesting that HbR (1) is more strongly correlated to the blood oxygen level dependent signal (Boas et al., 2004, 2014; Ferrari & Quaresima, 2012); (2) has greater spatial specificity than HbO (Noah et al., 2021; Zhang et al., 2016); (3) has been validated for speech-language tasks (Zhang et al., 2017); and (4) is less sensitive to systemic effects (e.g., heart rate, breathing, bloodflow) than HbO (Franceschini et al., 2003; Kirilina et al., 2012; Santosa et al., 2019; Scholkmann et al., 2013; Tachtsidis & Scholkmann, 2016; Zhang et al., 2016). Still, Figure S2 and Table S2 include the uncorrected whole-brain results for both chromophores.

**Figure 3.**
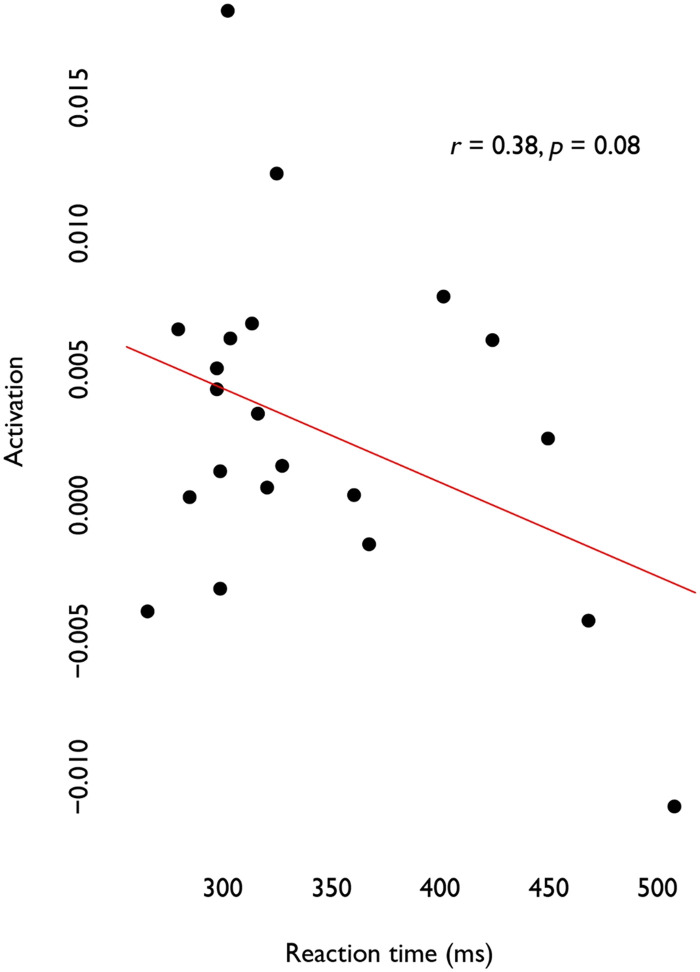
Correlation between activation and reaction time (ms) in R-DLPFC (approached significance). Deoxyhemoglobin (HbR) signals prior to sign reversal such that decreasing values reflect increases in activation strength.

The two within-group analyses that compared activation to rest provided some support that anticipated words are associated with increased activation (*p* = 0.02), whereas there was no change for unanticipated words (*p* = 0.82). Thus, it appears that prior to execution, anticipated words recruited the R-DLPFC ROI, whereas unanticipated words did not. Lastly, the correlation between reaction time and anticipation approached significance (*r* = 0.38, *p* = 0.08), indicating that for anticipated words, reaction time may increase as activation increases (Figure 3).

(2) *Stuttered vs. Fluent*: Activation in the R-DLPFC mask, using the same 5 s time window during the anticipation phase, was also greater for stuttered vs. fluent trials (*p* = 0.0039). See Figure S3 and Table S3 for uncorrected whole-brain results for HbR as well as HbO. Given that anticipation is strongly associated with stuttering (above), it is possible that the previous results for the anticipated vs. unanticipated contrast may be in part due to atypical planning associated with stuttering that is not necessarily related to anticipation. It was not possible to compare activation associated with *anticipated*/*stuttered*, *anticipated*/*fluent*, *unanticipated*/*stuttered*, and *unanticipated*/*fluent*, due to the unbalanced distribution of stuttering within participants (see Table 3) and the relatively limited number of trials. Importantly, however, 33.4% of unanticipated words were unambiguously stuttered, and 35.4% of anticipated words were unambiguously fluent (35.4%).

(3) *Stutterers vs. Controls*: Activation in the R-DLPFC mask, during the anticipation phase, was greater for stutterers compared to control speakers (*p* = 0.0442), irrespective of anticipation and stuttering. See Figure S4 and Table S4 for uncorrected whole-brain results for HbR and HbO.

(4) *Task vs. Rest (controls only)*: We did not find evidence that R-DLPFC was significantly activated during the “anticipation” phase in the control group (*p* = 0.1764). This may indicate that the area in the R-DLPFC mask was not recruited for speech planning, which is in line with previous accounts of speech motor control that do not attribute speech motor function to R-DLPFC.

(5) *Interactive vs. Alone*: Condition was not a variable of interest in the current study, but it was important to confirm that condition (interactive vs. alone) did not contribute to R-DLPFC overactivation. A significant difference between the interactive and alone conditions was not observed (*p* = 0.4826).

Significant differences in activation were not observed for anticipated vs. unanticipated words for the two control ROIs: L-DLPFC (*p* = 0.99); R-preCG (*p* = 0.95). The lack of significant differences in these areas provides evidence that the differences observed in the R-DLPFC ROI were due to the contrast (anticipated vs. unanticipated) and not to general differences associated with the stuttering brain or systemic artifact. In addition, significant activation differences were not observed between anticipated vs. unanticipated words in R-IFG (*p* = 0.75) or R-preSMA (*p* = 0.39).

#### Functional connectivity

Functional connectivity between the R-DLPFC and R-SMG was assessed using each node as a seed and target, and Holm-Bonferroni correction was applied for the two comparisons. Compared to unanticipated words, anticipated words were associated with lower intrinsic connectivity when R-DLPFC was the seed and R-SMG was the target (*t* = −2.89, *p* = 0.01), and also when R-SMG was the seed and R-DLPFC was the target (*t* = −2.06, *p* = 0.05). This reduction in functional connectivity between the R-DLPFC and R-SMG for anticipated relative to unanticipated words is taken as evidence of involvement of the FPN in stuttering anticipation.

## DISCUSSION

That stutterers anticipate overt stuttering events is well known, but the neural substrates of anticipation had not been studied previously. In this study, we used a novel, clinically inspired approach to identify anticipated and unanticipated words in a relatively large sample of adults who stutter. The words were produced in a delayed-response paradigm while neural signals were recorded with fNIRS. We identified R-DLPFC as a neural substrate of stuttering anticipation. A connectivity analysis was also conducted to explore whether the FPN, specifically the R-DLPFC and R-SMG, is associated with stuttering anticipation. Results are discussed in the context of theoretical accounts of stuttering anticipation, error-likelihood monitoring, and action-stopping. Our findings and potential limitations, as well as possible clinical implications, are also discussed in the following sections.

### Right Dorsolateral Prefrontal Cortex Underlies Stuttering Anticipation

The primary hypothesis was confirmed—anticipated words are associated with greater pre-execution activation in the R-DLPFC, compared to unanticipated words. This means that the production of words previously identified by participants as being difficult or likely to be stuttered, up-regulates activation in this area. It was also shown that stutterers exhibit greater activation than non-stutterers during this same time period, irrespective of anticipation and stuttering, and while anticipated words elicited activation in the R-DLPFC ROI in stutterers, unanticipated words of stutterers, and all words produced by controls, did not elicit activation in this area. Further, anticipated words were associated with longer reaction times, and there was some indication that as activation in R-DLPFC increased, so did reaction time (for anticipated words only). This extra time may be due to speakers delaying speech onset until the word can appear fluent to listeners, or “letting the stuttering pass,” which could be a function of the R-DLPFC. Our results provide some clarification of Kell et al. (2009), which found that stutterers exhibit greater activation than controls in the R-DLPFC, but that after therapy this is not the case, suggesting that therapy down-regulates an overactive R-DLPFC. Kell et al. (2009) also found that stutterers who reportedly “recovered” from stuttering after early childhood exhibited similar activation compared to controls in the R-DLPFC, suggesting that elevated activation in the R-DLPFC is a maladaptive response to stuttering. Our results suggest that the Kell et al. (2009) result was due to stuttering anticipation. It is important to highlight that we focused on longer- (vs. shorter-) term anticipation in this study, as stimuli were identified between 3 and 10 days prior to the fNIRS experiment. While it is possible that the neural processes for longer- and shorter-term anticipation overlap, many words identified as anticipated before the experiment were not stuttered during the experiment, indicating that they may not have been anticipated either, suggesting that there may be differences in the underlying processes related to longer- vs. shorter-term anticipation. Future studies can attempt to disentangle these types of anticipation to provide clarity on the time scales associated with anticipation.

Stuttered speech was also associated with elevated activation in R-DLPFC, calling into question whether atypical activation in this area was due to anticipation, or aberrant planning associated with stuttering. Given our novel approach, it was not possible to completely differentiate processes associated with anticipation and aberrant planning because we did not have enough power to make comparisons between *anticipated-stuttered*, *anticipated-fluent*, *unanticipated-stuttered*, *unanticipated-fluent* speech. fNIRS, like fMRI, relies on the slow hemodynamic response, which requires longer trials. With 80 trials already, each lasting at least 20 s, the experiment lasted approximately 30 min, which pushed the comfort threshold for participants. Together with the unpredictability of stuttering, this resulted in too few trials for the aforementioned analysis. However, there is reason to believe that activation differences were in fact driven by anticipation. First, a significant portion of anticipated/unanticipated words were fluent/stuttered, respectively. 33% of anticipated words were unambiguously fluent, whereas 33% of unanticipated words were unambiguously stuttered. Second, it is likely that anticipation was present throughout the 5 s anticipation phase, whereas it is unlikely that speech planning processes would comprise the entire 5 s anticipation phase. Third, we did not find evidence that non-stutterers activate R-DLPFC during the anticipation phase, suggesting that R-DLPFC does not play a primary role in speech planning. This is consistent with work on speech motor control or production models that do not include DLPFC as a part of the speech motor network (e.g., Forseth et al., 2021; Guenther, 2016; Sörös et al., 2006). Future work can tease these processes apart by obtaining enough trials to make the necessary statistical comparisons. It should also be noted, however, that due to the dynamic nature of both anticipation and stuttering, it may not be possible to completely differentiate these processes, especially because speech execution, or the possibility of it, is required to elicit anticipation in speakers.

### Error-Likelihood Monitoring

**Figure 4.**
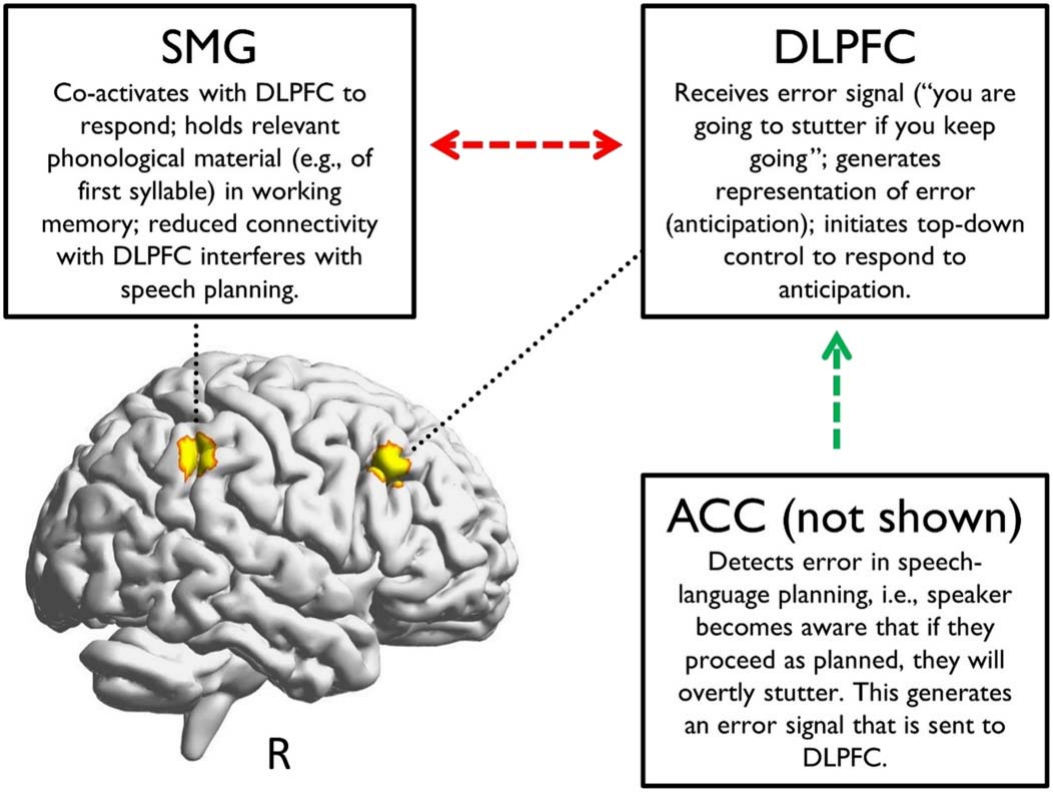
A model of stuttering anticipation. Anticipation develops via associative learning. Memory traces of associations form between overtly stuttered utterances and environmental/listener reactions. ACC detects upcoming stuttering via error-likelihood monitoring and signals R-DLPFC, which initiates cognitive control to respond to anticipation, thereby elevating activation in R-DLPFC. R-DLPFC initiates activation within the broader FPN to support the response. Atypical connection between R-DLPFC and areas within the FPN, such as SMG, impedes the speaker’s ability to adaptively respond to anticipation. ACC = anterior cingulate cortex; R-DLPFC = right dorsolateral prefrontal cortex; FPN = frontoparietal control network; R-SMG = right supramarginal gyrus.

Anticipation is likely driven by error-likelihood monitoring, which refers to the ability to predict errors based on prior experience making those errors. In their original account, Brown and Braver (2005) showed that ACC learns to predict the likelihood of errors, generating a “warning signal” to heighten readiness or initiate cognitive control in response to predicted errors. Arenas (2012, 2017) and Garcia-Barrera and Davidow (2015) extended the error-likelihood account to stuttering, whereby associative learning is the basis for anticipation. The speaker learns that some words/sounds are difficult, and when the speaker next says these words/sounds, they are primed to respond to upcoming stuttering. DLPFC works in concert with ACC to detect and respond to anticipated errors, such that ACC underlies the detection of errors in response to unintended outcomes, and subsequently generates error signals, whereas DLPFC generates representations of these errors including holding task-relevant information in working memory, and initiating subsequent actions (Alexander & Brown, 2015; Botvinick et al., 2001; Donoso et al., 2014; Holroyd & Yeung, 2012; Kolling et al., 2012). Interestingly, while Brown and Braver (2005) primarily focused on ACC, they also found error-likelihood effects in the R-DLPFC and R-SMG, which were less connected functionally for anticipated vs. unanticipated words in the current study. Thus, anticipation may destabilize the FPN, potentially reducing speakers’ control in responding to anticipation. We propose a model that extends the Arenas and Garcia-Barrera and Davidow accounts to the right FPN (see Figure 4). The ACC detects the error (i.e., anticipates) and subsequently generates an error signal that is sent to the R-DLPFC, which coupled with R-SMG, holds this information in working memory and initiates a response. Ultimately, we could not measure ACC due to imaging depth restrictions associated with fNIRS. Future work can test this proposal using fMRI.

### Action-stopping

A related interpretation involves the action-stopping network. Action-stopping has been studied primarily in the lab using the stop-signal task, in which participants are required to stop an initiated response when a signal occurs. Aron and colleagues (Aron, 2011; Wessel & Aron, 2017) proposed a prefrontal-basal ganglia-thalamocortical model of action-stopping in which prefrontal areas (R-IFG, R-preSMA) receive sensory information and subsequently produce a stop command, which is then transmitted to the subthalamic nucleus. The subthalamic nucleus then relays this information to the globus pallidus interna of the basal ganglia, which then inhibits the excitatory drive to motor cortex, i.e., to stop the action (Aron, 2011; Wessel & Aron, 2017). It has been proposed that this mechanism underlies stuttering by inducing a global inhibition response (over-suppression) that impedes the execution of successive motor programs (Arenas, 2017; Aron et al., 2014; Neef et al., 2018). Our results did not support this hypothesis—we did not find evidence of heightened activity in R-IFG or R-preSMA. This may be because the global inhibition account attempts to explain stuttering events at a speech motor control level, whereas anticipation occurs at a cognitive control level.

Disentangling reactive and proactive control, two forms of action-stopping, may provide clarity regarding the distinction between speech motor and cognitive control processes associated with stuttering. Reactive control is stimulus-driven and habitual or automatic, whereas proactive control is prospective and goal-directed (Hannah & Aron, 2021). Reactive control is supported by the hyperdirect cortico-subthalamic-pallidal-thalamo-cortical pathway, and includes superficial cortical structures, the R-IFG, and R-preSMA (Jahanshahi et al., 2015). This pathway is likely associated with the global inhibition that has been proposed to prevent the execution of successive motor programs (e.g., Arenas, 2017; Aron et al., 2014; Neef et al., 2018), i.e., to cause or trigger stuttering events. Proactive control, on the other hand, is regulated by the indirect fronto-striato-pallido-thalamo-cortical pathway, which includes the R-DLPFC, R-SMG, caudate, and thalamus (A. Chang et al., 2017; Chikazoe et al., 2009; Jahanshahi et al., 2015; Jahfari et al., 2010; Marek & Dosenbach, 2018; Smittenaar et al., 2013). Neef et al. (2018) proposed that proactive control may underlie responses to stuttering anticipation, which is supported by the current findings of overactivation in the R-DLPFC and reduced connectivity between R-DLPFC and R-SMG. Thus, while the indirect and hyperdirect pathways share neural circuitry, the hyperdirect pathway may underlie global suppression to prevent the succession of speech gestures (i.e., the stuttering event), whereas the indirect pathway seems to be related to how the speaker responds to *knowing* that the stuttering event is going to happen. The temporal dynamics associated with fNIRS as well as the slow hemodynamic response makes fNIRS a sub-optimal tool to test this hypothesis. Future studies could use fMRI and MEG to tease apart the specific spatial and temporal contributions (respectively) of both pathways, as well as interactions with the error monitoring system (including the ACC), to the manifestation of overt stuttering events and the speaker’s anticipation of them.

### Clinical Implications

The current work may inform neuromodulation techniques that have recently been applied to stuttering in clinical trials. Chesters et al. (2018) tested the impact of anodal transcranial direct current stimulation (tDCS) of left inferior frontal cortex on overt severity in 30 adult stutterers. They found that the treatment significantly reduced overt stuttering severity at a 1-week follow-up assessment, and at 6 weeks for reading but not conversational speech (Chesters et al., 2018). Garnett et al. (2019) tested the impact of anodal tDCS on overt severity in 14 adult stutterers. They did not find significant effects on overt stuttering severity, though they found that the atypically strong association between overt severity and right thalamocortical activity was attenuated, especially in severe stutterers. It may be that the modest effects reported to date are due to the focus on single areas, specifically in the speech network. Stuttering is not simply a speech motor control problem. Our data show that the cognitive and sensorimotor processes that underlie anticipation and subsequent overt stuttering elicit elevated activation in R-DLPFC, as well as potentially other areas within the FPN. Down-regulating R-DLPFC using tDCS and concurrently up-regulating, for example, left speech-language areas (premotor cortex/inferior frontal gyrus), which are typically under-active in speakers who stutter, could be the basis for clinical trials of the effects of tDCS in stuttering therapy.

Behaviorally, the anticipation of stuttering allows stutterers to disguise stuttering, such that there will be a discrepancy between what listeners hear or see and what speakers experience. While anticipating stuttering, the speaker may experience anxiety, fear, shame, or other cognitive responses, but the listener may not be privy to this information, creating misunderstanding between the speaker and the listener that could lead to negative listener perceptions of stuttering. For example, a listener might judge a speaker for looking “nervous” or for not being intelligent because they do not respond in a timely manner (as perceived by the listener). Understanding how the brain functions during anticipation could mitigate this discrepancy through increased understanding of the phenomenon and public awareness. This could also improve how SLPs work with people who stutter, particularly with respect to helping clients develop adaptive responses to anticipation. Developing such adaptive responses during therapy can be challenging, primarily because of the unobservable or “hidden” nature of anticipation. A brain-based understanding of anticipation may provide an entry point to begin discussing anticipation with their clients.

### Limitations

One limitation of this study is that the hypothesized effect in R-DLPFC—activation greater for anticipated vs. unanticipated words—was only evident based on the HbR signal, not HbO. For other motor tasks (e.g., finger tapping), effects are expected to be present for both chromophores. However, it is important to highlight that there is evidence that HbR may be a more reliable signal for speech tasks. For example, HbR is more strongly correlated to the blood oxygen level dependent signal compared to HbO (Boas et al., 2004, 2014; Ferrari & Quaresima, 2012), has greater spatial specificity than HbO (Noah et al., 2021; Zhang et al., 2016), and has been validated for speech-language tasks (Zhang et al., 2017). Further, HbR is less sensitive to systemic effects (e.g., heart rate, breathing, blood flow) than HbO (Franceschini et al., 2003; Kirilina et al., 2012; Santosa et al., 2019; Scholkmann et al., 2013; Tachtsidis & Scholkmann, 2016; Zhang et al., 2016), which may be relevant to stuttering because stuttering speakers may experience elevated anxiety/heart rate prior to speaking tasks. Neither anxiety nor autonomic data were collected in the current study, but either may have contributed to activation in R-DLPFC. Future studies could examine the impact of systemic effects on HbO and HbR, particularly for populations that may exhibit greater systemic effects such as anxiety. This would allow researchers both to determine whether systemic effects differentially impact HbO/HbR, and also to differentiate between anticipation and anxiety, to the extent that the latter is even possible.

Another limitation is that the design did not allow us to differentiate between the awareness of upcoming stuttering and the speaker’s response to the knowledge that upcoming speech will be stuttered (should they initiate their speech plan as intended). Thus, anticipation and *responding* to anticipation are conflated. Future work can address this limitation, for example, by testing the model of anticipation proposed herein, which postulates that the initial awareness of upcoming speech breakdown may occur in ACC, whereas the response to anticipation may be supported by R-DLPFC and the broader FPN. A third limitation is that anticipation was not measured during the actual experiment (e.g., by using a button press), and therefore there was no indication of the extent to which participants anticipated, if they anticipated at all. There were several reasons for this. First, we did not want to increase the length of the experiment, which was already nearing the participant comfort threshold. Identifying whether anticipation occurred could be difficult for some, if not all, participants, which would require additional time. Second, the act of identifying anticipation may alter neural responses. If identification comes before the trial, the act of identifying itself could alter the neural response. If identification occurs after the trial, responses may be biased based on whether the trial was overtly stuttered. One compromise could be to ask participants to identify the extent of anticipation for all words that were previously identified as anticipated just prior to the experiment. Participants could be presented with stimuli as they would during the actual experiment, which may help with identifying whether a word would be anticipated.

### Conclusion

This study determined that the R-DLPFC is a likely neural substrate of stuttering anticipation, and also that anticipation may be associated with reduced connectivity within the right hemisphere FPN. The results support accounts of error-likelihood monitoring and action-stopping and their association with stuttering events. Future investigations will benefit from adapting the current paradigm for use with fMRI and MEG to determine the relationships between error-likelihood monitoring, action-stopping, and stuttering, and whether functional connectivity within the FPN and related networks (salience, cingulo-opercular) is weaker for anticipated words. It will also be critical to study children who stutter so that the developmental bases of anticipation can be determined. Finally, results from this study may inform clinical trials that test the efficacy of neuromodulation in stuttering therapy, particularly by focusing not only on speech motor control networks, but also cognitive control and related networks.

## ACKNOWLEDGMENTS

The authors thank Drs. Joan Orpella, Adam Buchwald, and Christian A. Kell for reading earlier versions of this manuscript. Most importantly, the authors thank the participants. The science of stuttering will not advance without the willing participation of speakers who stutter, and we are greatly appreciative of their commitment and time.

## FUNDING INFORMATION

Eric S. Jackson, National Institute on Deafness and Other Communication Disorders (https://dx.doi.org/10.13039/100000055), Award ID: R21DC017821. Swethasri Dravida, National Institutes of Health (https://dx.doi.org/10.13039/100000002), Award ID: T32GM007205. Swethasri Dravida, National Institutes of Health (https://dx.doi.org/10.13039/100000002), Award ID: F30MH116626. Joy Hirsch, National Institute of Mental Health, Award ID: R01MH111629. Joy Hirsch, National Institute of Mental Health, Award ID: R01MH119430.

## AUTHOR CONTRIBUTIONS

**Eric S. Jackson**: Conceptualization: Lead; Formal analysis: Equal; Funding acquisition: Lead; Investigation: Lead; Methodology: Lead; Project administration: Lead; Writing: Lead. **Swethasri Dravida**: Conceptualization: Supporting; Formal analysis: Equal; Investigation: Supporting; Methodology: Supporting; Writing: Supporting. **Xian Zhang**: Conceptualization: Supporting; Formal analysis: Equal; Investigation: Supporting; Methodology: Supporting; Software: Lead; Writing: Supporting. **J. Adam Noah**: Conceptualization: Supporting; Formal analysis: Supporting; Investigation: Supporting; Methodology: Supporting; Writing: Supporting. **Vincent Gracco**: Conceptualization: Supporting; Methodology: Supporting; Supervision: Supporting; Writing: Supporting. **Joy Hirsch**: Conceptualization: Supporting; Methodology: Supporting; Supervision: Supporting; Writing: Supporting.

## References

[bib1] Alexander, W. H., & Brown, J. W. (2015). Hierarchical error representation: A computational model of anterior cingulate and dorsolateral prefrontal cortex. Neural Computation, 27(11), 2354–2410. 10.1162/NECO_a_00779, 26378874

[bib2] Anderson, J. D., & Ofoe, L. C. (2019). The role of executive function in developmental stuttering. Seminars in Speech and Language, 40(4), 305–319. 10.1055/s-0039-1692965, 31311055 PMC6910129

[bib3] Arenas, R. M. (2012). The role of anticipation and an adaptive monitoring system in stuttering: A theoretical and experimental investigation [Unpublished doctoral dissertation]. University of Iowa.

[bib4] Arenas, R. M. (2017). Conceptualizing and investigating the contextual variability of stuttering: The speech and monitoring interaction (SAMI) framework. Speech, Language and Hearing, 20(1), 15–28. 10.1080/2050571X.2016.1221877

[bib5] Aron, A. R. (2011). From reactive to proactive and selective control: Developing a richer model for stopping inappropriate responses. Biological Psychiatry, 69(12), e55–e68. 10.1016/j.biopsych.2010.07.024, 20932513 PMC3039712

[bib6] Aron, A. R., Robbins, T. W., & Poldrack, R. A. (2014). Inhibition and the right inferior frontal cortex: One decade on. Trends in Cognitive Sciences, 18(4), 177–185. 10.1016/j.tics.2013.12.003, 24440116

[bib7] Barton, K. (2020). Mu-MIn: Multi-model inference (R Package Version 0.12. 2/r18. 2009) [Computer software]. https://CRAN.R-project.org/

[bib8] Bates, D. M., Maechler, M., Bolker, B., & Walker, S. (2014). lme4: Linear mixed-effects models using ‘Eigen’ and S4 (R package version 1.1-7) [Computer software]. https://CRAN.R-project.org/package=lme4

[bib9] Belyk, M., Kraft, S. J., & Brown, S. (2015). Stuttering as a trait or state: An ALE meta-analysis of neuroimaging studies. European Journal of Neuroscience, 41(2), 275–284. 10.1111/ejn.12765, 25350867 PMC13140564

[bib10] Belyk, M., Kraft, S. J., & Brown, S. (2017). Stuttering as a trait or a state revisited: Motor system involvement in persistent developmental stuttering. European Journal of Neuroscience, 45(4), 622–624. 10.1111/ejn.13512, 28191730

[bib11] Boas, D. A., Dale, A. M., & Franceschini, M. A. (2004). Diffuse optical imaging of brain activation: Approaches to optimizing image sensitivity, resolution, and accuracy. NeuroImage, 23(S1), S275–S288. 10.1016/j.neuroimage.2004.07.011, 15501097

[bib12] Boas, D. A., Elwell, C. E., Ferrari, M., & Taga, G. (2014). Twenty years of functional near-infrared spectroscopy: Introduction for the special issue. NeuroImage, 85(Pt. 1), 1–5. 10.1016/j.neuroimage.2013.11.033, 24321364

[bib13] Botvinick, M. M., Braver, T. S., Barch, D. M., Carter, C. S., & Cohen, J. D. (2001). Conflict monitoring and cognitive control. Psychological Review, 108(3), 624–652. 10.1037/0033-295X.108.3.624, 11488380

[bib14] Bowers, A., Saltuklaroglu, T., & Kalinowski, J. (2012). Autonomic arousal in adults who stutter prior to various reading tasks intended to elicit changes in stuttering frequency. International Journal of Psychophysiology, 83(1), 45–55. 10.1016/j.ijpsycho.2011.09.021, 22044550

[bib15] Braun, A. R., Varga, M., Stager, S., Schulz, G., Selbie, S., Maisog, J. M., Carson, R. E., & Ludlow, C. L. (1997). Altered patterns of cerebral activity during speech and language production in developmental stuttering. An H2(15)O positron emission tomography study. Brain, 120(5), 761–784. 10.1093/brain/120.5.761, 9183248

[bib16] Brett, M., Anton, J.-L., Valabregue, R., & Poline, J.-B. (2002, June 2–6). Region of interest analysis using an SPM toolbox [Paper presentation]. The 8th International Conference on Functional Mapping of the Human Brain, Sendai, Japan.

[bib17] Brown, J. W., & Braver, T. S. (2005). Learned predictions of error likelihood in the anterior cingulate cortex. Science, 307(5712), 1118–1121. 10.1126/science.1105783, 15718473

[bib18] Budde, K. S., Barron, D. S., & Fox, P. T. (2014). Stuttering, induced fluency, and natural fluency: A hierarchical series of activation likelihood estimation meta-analyses. Brain and Language, 139, 99–107. 10.1016/j.bandl.2014.10.002, 25463820 PMC4405378

[bib19] Chang, A., Ide, J. S., Li, H.-H., Chen, C.-C., & Li, C.-S. R. (2017). Proactive control: Neural oscillatory correlates of conflict anticipation and response slowing. eNeuro, 4(3), Article 0061-17.2017. 10.1523/ENEURO.0061-17.2017, 28560315 PMC5446487

[bib20] Chang, S.-E., Horwitz, B., Ostuni, J., Reynolds, R., & Ludlow, C. L. (2011). Evidence of left inferior frontal–premotor structural and functional connectivity deficits in adults who stutter. Cerebral Cortex, 21(11), 2507–2518. 10.1093/cercor/bhr028, 21471556 PMC3183422

[bib21] Chesters, J., Möttönen, R., & Watkins, K. E. (2018). Transcranial direct current stimulation over left inferior frontal cortex improves speech fluency in adults who stutter. Brain, 141(4), 1161–1171. 10.1093/brain/awy011, 29394325 PMC6019054

[bib22] Chikazoe, J., Jimura, K., Hirose, S., Yamashita, K., Miyashita, Y., & Konishi, S. (2009). Preparation to inhibit a response complements response inhibition during performance of a stop-signal task. Journal of Neuroscience, 29(50), 15870–15877. 10.1523/JNEUROSCI.3645-09.2009, 20016103 PMC6666181

[bib23] De Nil, L. F., Kroll, R. M., Kapur, S., & Houle, S. (2000). A positron emission tomography study of silent and oral single word reading in stuttering and nonstuttering adults. Journal of Speech, Language and Hearing Research, 43(4), 1038–1053. 10.1044/jslhr.4304.1038, 11386470

[bib24] De Nil, L. F., Kroll, R. M., Lafaille, S. J., & Houle, S. (2004). A positron emission tomography study of short- and long-term treatment effects on functional brain activation in adults who stutter. Journal of Fluency Disorders, 28(4), 357–380. 10.1016/j.jfludis.2003.07.002, 14643070

[bib25] Descorbeth, O., Zhang, X., Noah, J. A., & Hirsch, J. (2020). Neural processes for live pro-social dialogue between dyads with socioeconomic disparity. Social Cognitive and Affective Neuroscience, 15(8), 875–887. 10.1093/scan/nsaa120, 32879986 PMC7543936

[bib26] D’Esposito, M., & Postle, B. R. (2002). The neural basis of working memory storage, rehearsal, and control processes: Evidence from patient and functional magnetic resonance imaging studies. In L. R. Squire & D. L. Schacter (Eds.), Neuropsychology of memory (pp. 215–224). Guilford Press.

[bib27] Donoso, M., Collins, A. G., & Koechlin, E. (2014). Foundations of human reasoning in the prefrontal cortex. Science, 344(6191), 1481–1486. 10.1126/science.1252254, 24876345

[bib28] Eggebrecht, A. T., White, B. R., Ferradal, S. L., Chen, C., Zhan, Y., Snyder, A. Z., Dehghani, H., & Culver, J. P. (2012). A quantitative spatial comparison of high-density diffuse optical tomography and fMRI cortical mapping. NeuroImage, 61(4), 1120–1128. 10.1016/j.neuroimage.2012.01.124, 22330315 PMC3581336

[bib29] Ferradal, S. L., Eggebrecht, A. T., Hassanpour, M., Snyder, A. Z., & Culver, J. P. (2014). Atlas-based head modeling and spatial normalization for high-density diffuse optical tomography: In vivo validation against fMRI. NeuroImage, 85(1), 117–126. 10.1016/j.neuroimage.2013.03.069, 23578579 PMC4433751

[bib30] Ferrari, M., & Quaresima, V. (2012). A brief review on the history of human functional near-infrared spectroscopy (fNIRS) development and fields of application. NeuroImage, 63(2), 921–935. 10.1016/j.neuroimage.2012.03.049, 22510258

[bib31] Forseth, K. J., Pitkow, X., Fischer-Baum, S., & Tandon, N. (2021). What the brain does as we speak. BioRxiv. 10.1101/2021.02.05.429841

[bib32] Fox, P. T., Ingham, R. J., Ingham, J. C., Zamarripa, F., Xiong, J.-H., & Lancaster, J. L. (2000). Brain correlates of stuttering and syllable production: A PET performance-correlation analysis. Brain, 123(10), 1985–2004. 10.1093/brain/123.10.1985, 11004117

[bib33] Franceschini, M. A., Fantini, S., Thompson, J. H., Culver, J. P., & Boas, D. A. (2003). Hemodynamic evoked response of the sensorimotor cortex measured noninvasively with near-infrared optical imaging. Psychophysiology, 40(4), 548–560. 10.1111/1469-8986.00057, 14570163 PMC3786740

[bib34] Friston, K. J., Harrison, L., & Penny, W. (2003). Dynamic causal modelling. NeuroImage, 19(4), 1273–1302. 10.1016/S1053-8119(03)00202-7, 12948688

[bib35] Friston, K. J., Holmes, A. P., Poline, J. B., Grasby, P. J., Williams, S. C. R., Frackowiak, R. S., & Turner, R. (1995). Analysis of fMRI time-series revisited. NeuroImage, 2(1), 45–53. 10.1006/nimg.1995.1007, 9343589

[bib36] Friston, K. J., Holmes, A. P., Worsley, K. J., Poline, J.-P., Frith, C. D., & Frackowiak, R. S. (1994). Statistical parametric maps in functional imaging: A general linear approach. Human Brain Mapping, 2(4), 189–210. 10.1002/hbm.460020402

[bib37] Garcia-Barrera, M. A., & Davidow, J. H. (2015). Anticipation in stuttering: A theoretical model of the nature of stutter prediction. Journal of Fluency Disorders, 44, 1–15. 10.1016/j.jfludis.2015.03.002, 25841698

[bib38] Garnett, E. O., Chow, H. M., Choo, A. L., & Chang, S.-E. (2019). Stuttering severity modulates effects of non-invasive brain stimulation in adults who stutter. Frontiers in Human Neuroscience, 13, Article 411. 10.3389/fnhum.2019.00411, 31824276 PMC6881273

[bib39] Goldman-Rakic, P. S. (1988). Topography of cognition: Parallel distributed networks in primate association cortex. Annual Review of Neuroscience, 11(1), 137–156. 10.1146/annurev.ne.11.030188.001033, 3284439

[bib40] Guenther, F. H. (2016). Neural control of speech. MIT Press. 10.7551/mitpress/10471.001.0001

[bib41] Hannah, R., & Aron, A. R. (2021). Towards real-world generalizability of a circuit for action-stopping. Nature Reviews Neuroscience, 22(9), 538–552. 10.1038/s41583-021-00485-1, 34326532 PMC8972073

[bib42] Hirsch, J., Noah, J. A., Zhang, X., Dravida, S., & Ono, Y. (2018). A cross-brain neural mechanism for human-to-human verbal communication. Social Cognitive and Affective Neuroscience, 13(9), 907–920. 10.1093/scan/nsy070, 30137601 PMC6137318

[bib43] Hirsch, J., Tiede, M., Zhang, X., Noah, J. A., Salama-Manteau, A., & Biriotti, M. (2021). Interpersonal agreement and disagreement during face-to-face dialogue: An fNIRS investigation. Frontiers in Human Neuroscience, 14, Article 606397. 10.3389/fnhum.2020.606397, 33584223 PMC7874076

[bib44] Hirsch, J., Zhang, X., Noah, J. A., & Ono, Y. (2017). Frontal temporal and parietal systems synchronize within and across brains during live eye-to-eye contact. NeuroImage, 157, 314–330. 10.1016/j.neuroimage.2017.06.018, 28619652 PMC5863547

[bib45] Holroyd, C. B., & Yeung, N. (2012). Motivation of extended behaviors by anterior cingulate cortex. Trends in Cognitive Sciences, 16(2), 122–128. 10.1016/j.tics.2011.12.008, 22226543

[bib46] Jackson, E. S., Gerlach, H., Rodgers, N. H., & Zebrowski, P. M. (2018). My client knows that he’s about to stutter: How can we address stuttering anticipation during therapy with young people who stutter? Seminars in Speech and Language, 39, 356–370. 10.1055/s-0038-1667164, 30142646

[bib47] Jackson, E. S., Gracco, V., & Zebrowski, P. M. (2020). Eliciting stuttering in laboratory contexts. Journal of Speech, Language, and Hearing Research, 63(1), 143–150. 10.1044/2019_JSLHR-S-19-0173, 31835000 PMC7213478

[bib48] Jackson, E. S., Rodgers, N. H., & Rodgers, D. B. (2019). An exploratory factor analysis of action responses to stuttering anticipation. Journal of Fluency Disorders, 60, 1–10. 10.1016/j.jfludis.2019.03.001, 30875585

[bib49] Jackson, E. S., Yaruss, J. S., Quesal, R. W., Terranova, V., & Whalen, D. H. (2015). Responses of adults who stutter to the anticipation of stuttering. Journal of Fluency Disorders, 45, 38–51. 10.1016/j.jfludis.2015.05.002, 26065618 PMC4728710

[bib50] Jahanshahi, M., Obeso, I., Rothwell, J. C., & Obeso, J. A. (2015). A fronto–striato–subthalamic–pallidal network for goal-directed and habitual inhibition. Nature Reviews Neuroscience, 16(12), 719–732. 10.1038/nrn4038, 26530468

[bib51] Jahfari, S., Stinear, C. M., Claffey, M., Verbruggen, F., & Aron, A. R. (2010). Responding with restraint: What are the neurocognitive mechanisms? Journal of Cognitive Neuroscience, 22(7), 1479–1492. 10.1162/jocn.2009.21307, 19583473 PMC2952035

[bib52] Kell, C. A., Neumann, K., von Kriegstein, K., Posenenske, C., von Gudenberg, A. W., Euler, H., & Giraud, A.-L. (2009). How the brain repairs stuttering. Brain, 132(10), 2747–2760. 10.1093/brain/awp185, 19710179

[bib53] Kirilina, E., Jelzow, A., Heine, A., Niessing, M., Wabnitz, H., Brühl, R., Ittermann, B., Jacobs, A. M., & Tachtsidis, I. (2012). The physiological origin of task-evoked systemic artefacts in functional near infrared spectroscopy. NeuroImage, 61(1), 70–81. 10.1016/j.neuroimage.2012.02.074, 22426347 PMC3348501

[bib54] Knott, J. R., Johnson, W., & Webster, M. J. (1937). Studies in the psychology of stuttering: II A quantitative evaluation of expectation of stuttering in relation to the occurrence of stuttering. Journal of Speech Disorders, 2(1), 20–22. 10.1044/jshd.0201.20

[bib55] Koechlin, E., Ody, C., & Kouneiher, F. (2003). The architecture of cognitive control in the human prefrontal cortex. Science, 302(5648), 1181–1185. 10.1126/science.1088545, 14615530

[bib56] Kolling, N., Behrens, T. E., Mars, R. B., & Rushworth, M. F. (2012). Neural mechanisms of foraging. Science, 336(6077), 95–98. 10.1126/science.1216930, 22491854 PMC3440844

[bib57] Krall, E. A., & Dawson-Hughes, B. (1993). Heritable and life-style determinants of bone mineral density. Journal of Bone and Mineral Research, 8(1), 1–9. 10.1002/jbmr.5650080102, 8427042

[bib58] Kuznetsova, A., Brockhoff, P. B., & Christensen, R. H. B. (2017). lmerTest package: Tests in linear mixed effects models. Journal of Statistical Software, 82(13), 1–26. 10.18637/jss.v082.i13

[bib59] Lancaster, J. L., Woldorff, M. G., Parsons, L. M., Liotti, M., Freitas, C. S., Rainey, L., Kochunov, P. V., Nickerson, D., Mikiten, S. A., & Fox, P. T. (2000). Automated Talairach atlas labels for functional brain mapping. Human Brain Mapping, 10(3), 120–131. 10.1002/1097-0193(200007)10:3<120::AID-HBM30>3.0.CO;2-8, 10912591 PMC6871915

[bib60] Lu, C., Ning, N., Peng, D., Ding, G., Li, K., Yang, Y., & Lin, C. (2009). The role of large-scale neural interactions for developmental stuttering. Neuroscience, 161(4), 1008–1026. 10.1016/j.neuroscience.2009.04.020, 19364522

[bib61] MacDonald, A. W., Cohen, J. D., Stenger, V. A., & Carter, C. S. (2000). Dissociating the role of the dorsolateral prefrontal and anterior cingulate cortex in cognitive control. Science, 288(5472), 1835–1838. 10.1126/science.288.5472.1835, 10846167

[bib62] Marek, S., & Dosenbach, N. U. (2018). The frontoparietal network: Function, electrophysiology, and importance of individual precision mapping. Dialogues in Clinical Neuroscience, 20(2), 133–140. 10.31887/DCNS.2018.20.2/smarek, 30250390 PMC6136121

[bib63] Matcher, S. J., Elwell, C. E., Cooper, C. E., Cope, M., & Delpy, D. T. (1995). Performance comparison of several published tissue near-infrared spectroscopy algorithms. Analytical Biochemistry, 227(1), 54–68. 10.1006/abio.1995.1252, 7668392

[bib64] McHugh, M. L. (2012). Interrater reliability: The kappa statistic. Biochemia Medica, 22(3), 276–282. 10.11613/BM.2012.031, 23092060 PMC3900052

[bib65] McLaren, D. G., Ries, M. L., Xu, G., & Johnson, S. C. (2012). A generalized form of context-dependent psychophysiological interactions (gPPI): A comparison to standard approaches. NeuroImage, 61(4), 1277–1286. 10.1016/j.neuroimage.2012.03.068, 22484411 PMC3376181

[bib66] Menon, V., & D’Esposito, M. (2021). The role of PFC networks in cognitive control and executive function. Neuropsychopharmacology, 47, 1–14. 10.1038/s41386-021-01152-w. 34408276 PMC8616903

[bib67] Mersov, A., Cheyne, D., Jobst, C., & De Nil, L. (2018). A preliminary study on the neural oscillatory characteristics of motor preparation prior to dysfluent and fluent utterances in adults who stutter. Journal of Fluency Disorders, 55, 145–155. 10.1016/j.jfludis.2017.05.003, 28577876

[bib68] Mesulam, M. M. (1998). From sensation to cognition. Brain, 121(6), 1013–1052. 10.1093/brain/121.6.1013, 9648540

[bib69] Milisen, R. (1938). Frequency of stuttering with anticipation of stuttering controlled. Journal of Speech Disorders, 3(4), 207–214. 10.1044/jshd.0304.207

[bib70] Miller, E. K. (2000). The prefrontal cortex and cognitive control. Nature Reviews Neuroscience, 1(1), 59–65. 10.1038/35036228, 11252769

[bib71] Neef, N. E., Anwander, A., Bütfering, C., Schmidt-Samoa, C., Friederici, A. D., Paulus, W., & Sommer, M. (2018). Structural connectivity of right frontal hyperactive areas scales with stuttering severity. Brain, 141(1), 191–204. 10.1093/brain/awx316, 29228195 PMC5837552

[bib72] Neef, N. E., Bütfering, C., Anwander, A., Friederici, A. D., Paulus, W., & Sommer, M. (2016). Left posterior-dorsal area 44 couples with parietal areas to promote speech fluency, while right area 44 activity promotes the stopping of motor responses. NeuroImage, 142, 628–644. 10.1016/j.neuroimage.2016.08.030, 27542724

[bib73] Neumann, K., Euler, H. A., von Gudenberg, A. W., Giraud, A.-L., Lanfermann, H., Gall, V., & Preibisch, C. (2004). The nature and treatment of stuttering as revealed by fMRI: A within- and between-group comparison. Journal of Fluency Disorders, 28(4), 381–410. 10.1016/j.jfludis.2003.07.003, 14643071

[bib74] Neumann, K., Preibisch, C., Euler, H. A., von Gudenberg, A. W., Lanfermann, H., Gall, V., & Giraud, A.-L. (2005). Cortical plasticity associated with stuttering therapy. Journal of Fluency Disorders, 30(1), 23–39. 10.1016/j.jfludis.2004.12.002, 15769497

[bib75] Niendam, T. A., Laird, A. R., Ray, K. L., Dean, Y. M., Glahn, D. C., & Carter, C. S. (2012). Meta-analytic evidence for a superordinate cognitive control network subserving diverse executive functions. Cognitive, Affective, & Behavioral Neuroscience, 12(2), 241–268. 10.3758/s13415-011-0083-5, 22282036 PMC3660731

[bib76] Noah, J. A., Zhang, X., Dravida, S., DiCocco, C., Suzuki, T., Aslin, R. N., Tachtsidis, I., & Hirsch, J. (2021). Comparison of short-channel separation and spatial domain filtering for removal of non-neural components in functional near-infrared spectroscopy signals. Neurophotonics, 8(1), Article 015004. 10.1117/1.NPh.8.1.015004, 33598505 PMC7881368

[bib77] Okada, E., & Delpy, D. T. (2003). Near-infrared light propagation in an adult head model. II. Effect of superficial tissue thickness on the sensitivity of the near-infrared spectroscopy signal. Applied Optics, 42(16), 2915–2921. 10.1364/AO.42.002915, 12790440

[bib78] Okamoto, M., & Dan, I. (2005). Automated cortical projection of head-surface locations for transcranial functional brain mapping. NeuroImage, 26(1), 18–28. 10.1016/j.neuroimage.2005.01.018, 15862201

[bib79] Preibisch, C., Neumann, K., Raab, P., Euler, H. A., von Gudenberg, A. W., Lanfermann, H., & Giraud, A.-L. (2003). Evidence for compensation for stuttering by the right frontal operculum. NeuroImage, 20(2), 1356–1364. 10.1016/S1053-8119(03)00376-8, 14568504

[bib80] R Core Team. (2014). R: A language and environment for statistical computing [Computer software]. R Foundation for Statistical Computing. https://www.R-project.org/

[bib81] Ridderinkhof, K. R., van den Wildenberg, W. P., Segalowitz, S. J., & Carter, C. S. (2004). Neurocognitive mechanisms of cognitive control: The role of prefrontal cortex in action selection, response inhibition, performance monitoring, and reward-based learning. Brain and Cognition, 56(2), 129–140. 10.1016/j.bandc.2004.09.016, 15518930

[bib82] Rorden, C., & Brett, M. (2000). Stereotaxic display of brain lesions. Behavioural Neurology, 12(4), Article 421719. 10.1155/2000/421719, 11568431

[bib83] Santosa, H., Fishburn, F., Zhai, X., & Huppert, T. J. (2019). Investigation of the sensitivity-specificity of canonical- and deconvolution-based linear models in evoked functional near-infrared spectroscopy. Neurophotonics, 6(2), Article 025009. 10.1117/1.NPh.6.2.025009, 31172019 PMC6541797

[bib84] Scholkmann, F., Gerber, U., Wolf, M., & Wolf, U. (2013). End-tidal CO_2_: An important parameter for a correct interpretation in functional brain studies using speech tasks. NeuroImage, 66, 71–79. 10.1016/j.neuroimage.2012.10.025, 23099101

[bib85] Smittenaar, P., Guitart-Masip, M., Lutti, A., & Dolan, R. J. (2013). Preparing for selective inhibition within frontostriatal loops. Journal of Neuroscience, 33(46), 18087–18097. 10.1523/JNEUROSCI.2167-13.2013, 24227719 PMC3828462

[bib86] Sörös, P., Guttman Sokoloff, L., Bose, A., McIntosh, A. R., Graham, S. J., & Stuss, D. T. (2006). Clustered functional MRI of overt speech production. NeuroImage, 32(1), 376–387. 10.1016/j.neuroimage.2006.02.046, 16631384

[bib87] Tachtsidis, I., & Scholkmann, F. (2016). False positives and false negatives in functional near-infrared spectroscopy: Issues, challenges, and the way forward. Neurophotonics, 3(3), Article 031405. 10.1117/1.NPh.3.3.031405, 27054143 PMC4791590

[bib88] Tichenor, S. E., & Yaruss, J. S. (2019). Repetitive negative thinking, temperament, and adverse impact in adults who stutter. American Journal of Speech-Language Pathology, 29(1), 201–215. 10.1044/2019_AJSLP-19-00077, 31846585

[bib89] Toyomura, A., Fujii, T., Yokosawa, K., & Kuriki, S. (2018). Speech disfluency-dependent amygdala activity in adults who stutter: Neuroimaging of interpersonal communication in MRI scanner environment. Neuroscience, 374, 144–154. 10.1016/j.neuroscience.2018.01.037, 29378280

[bib90] Van Riper, C. (1936). Study of the thoracic breathing of stutterers during expectancy and occurrence of stuttering spasm. Journal of Speech Disorders, 1(3), 61–72. 10.1044/jshd.0103.61

[bib91] Vassena, E., Gerrits, R., Demanet, J., Verguts, T., & Siugzdaite, R. (2019). Anticipation of a mentally effortful task recruits dorsolateral prefrontal cortex: An fNIRS validation study. Neuropsychologia, 123, 106–115. 10.1016/j.neuropsychologia.2018.04.033, 29705065

[bib92] Wessel, J. R., & Aron, A. R. (2017). On the globality of motor suppression: Unexpected events and their influence on behavior and cognition. Neuron, 93(2), 259–280. 10.1016/j.neuron.2016.12.013, 28103476 PMC5260803

[bib93] Wingate, M. E. (1975). Expectancy as basically a short-term process. Journal of Speech and Hearing Research, 18(1), 31–42. 10.1044/jshr.1801.31, 1127907

[bib94] Wymbs, N. F., Ingham, R. J., Ingham, J. C., Paolini, K. E., & Grafton, S. T. (2013). Individual differences in neural regions functionally related to real and imagined stuttering. Brain and Language, 124(2), 153–164. 10.1016/j.bandl.2012.11.013, 23333668 PMC3625940

[bib95] Xia, M., Wang, J., & He, Y. (2013). BrainNet Viewer: A network visualization tool for human brain connectomics. PLOS ONE, 8(7), Article e68910. 10.1371/journal.pone.0068910, 23861951 PMC3701683

[bib96] Yairi, E., & Ambrose, N. (1992). A longitudinal study of stuttering in children. Journal of Speech, Language, and Hearing Research, 35(4), 755–760. 10.1044/jshr.3504.755, 1405530

[bib97] Ye, J. C., Tak, S., Jang, K. E., Jung, J., & Jang, J. (2009). NIRS-SPM: Statistical parametric mapping for near-infrared spectroscopy. NeuroImage, 44(2), 428–447. 10.1016/j.neuroimage.2008.08.036, 18848897

[bib98] Zhang, X., Noah, J. A., Dravida, S., & Hirsch, J. (2017). Signal processing of functional NIRS data acquired during overt speaking. Neurophotonics, 4(4), Article 041409. 10.1117/1.NPh.4.4.041409, 28924564 PMC5592780

[bib99] Zhang, X., Noah, J. A., & Hirsch, J. (2016). Separation of the global and local components in functional near-infrared spectroscopy signals using principal component spatial filtering. Neurophotonics, 3(1), Article 015004. 10.1117/1.NPh.3.1.015004, 26866047 PMC4742567

