# Supplementary material for: Activation in Right Dorsolateral Prefrontal Cortex Underlies Stuttering Anticipation
Source: Neurobiol Lang (Camb). 2022 Aug 17;3(3):469–94. doi: 10.1162/nol_a_00073 (PMC10158639; doi:10.1162/nol_a_00073)
Supplement: Supplementary file 1 [file nol-3-3-469-s001.docx]

SUPPLEMENTARY MATERIAL

Supplementary Figure 1. Right (R) and left (L) hemispheres of rendered brains illustrate average locations (red circles) for the 134 channels per participant. Montreal Neurological Institute (MNI) coordinates were determined by digitizing the locations of the optodes in relation to the 10–20 system based on conventional landmarks. See Supplementary Table 1 for group median coordinates, anatomical regions, and atlas-based probabilities for each channel.


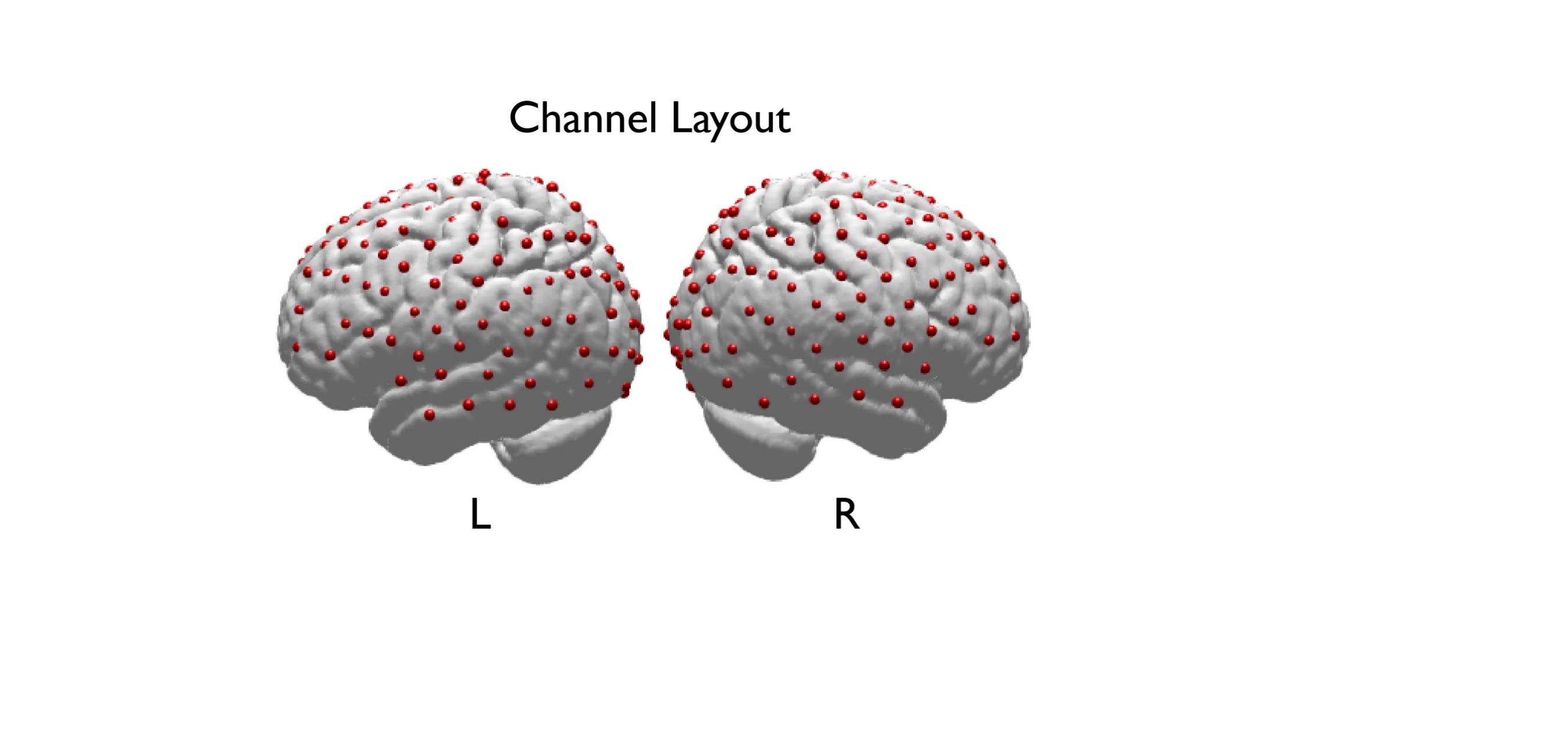


Supplementary Table 1. Group median coordinates, atlas-based probabilities, Brodmann’s areas, and anatomical regions for each channel. Montreal Neurological Institute (MNI) coordinates for each of the 134 channels per participant were determined by digitizing the locations of the optodes in relation to the 10-20 system based on conventional landmarks.

| Channel number | MNI coordinates | | | Probability | BA | Anatomical region |
| --- | --- | --- | --- | --- | --- | --- |
|  | X | Y | Z |  |  |  |
| 1 | -37 | 61 | 9 | 0.97 | 10 | Frontopolar area |
|  |  |  |  | 0.03 | 46 | Dorsolateral prefrontal cortex |
| 2 | 38 | 63 | 10 | 1 | 10 | Frontopolar area |
| 3 | -26 | 62 | 24 | 0.96 | 10 | Frontopolar area |
|  |  |  |  | 0.04 | 9 | Dorsolateral prefrontal cortex |
| 4 | 26 | 64 | 26 | 0.94 | 10 | Frontopolar area |
|  |  |  |  | 0.06 | 9 | Dorsolateral prefrontal cortex |
| 5 | -13 | 58 | 39 | 0.83 | 9 | Dorsolateral prefrontal cortex |
|  |  |  |  | 0.14 | 10 | Frontopolar area |
|  |  |  |  | 0.03 | 8 | Includes Frontal eye fields |
| 6 | 13 | 59 | 39 | 0.81 | 9 | Dorsolateral prefrontal cortex |
|  |  |  |  | 0.17 | 10 | Frontopolar area |
|  |  |  |  | 0.02 | 8 | Includes Frontal eye fields |
| 7 | -23 | 48 | 44 | 0.54 | 9 | Dorsolateral prefrontal cortex |
|  |  |  |  | 0.46 | 8 | Includes Frontal eye fields |
| 8 | -1 | 51 | 48 | 0.7 | 8 | Includes Frontal eye fields |
|  |  |  |  | 0.3 | 9 | Dorsolateral prefrontal cortex |
| 9 | 22 | 50 | 46 | 0.58 | 8 | Includes Frontal eye fields |
|  |  |  |  | 0.42 | 9 | Dorsolateral prefrontal cortex |
| 10 | -49 | 37 | 29 | 0.88 | 46 | Dorsolateral prefrontal cortex |
|  |  |  |  | 0.12 | 9 | Dorsolateral prefrontal cortex |
| 11 | -33 | 38 | 46 | 0.7 | 8 | Includes Frontal eye fields |
|  |  |  |  | 0.3 | 9 | Dorsolateral prefrontal cortex |
| 12 | -12 | 41 | 55 | 0.98 | 8 | Includes Frontal eye fields |
|  |  |  |  | 0.02 | 6 | Pre-Motor and Supplementary Motor Cortex |
| 13 | 12 | 42 | 56 | 0.97 | 8 | Includes Frontal eye fields |
|  |  |  |  | 0.03 | 6 | Pre-Motor and Supplementary Motor Cortex |
| 14 | 33 | 40 | 47 | 0.77 | 8 | Includes Frontal eye fields |
|  |  |  |  | 0.23 | 9 | Dorsolateral prefrontal cortex |
| 15 | 50 | 39 | 32 | 0.66 | 46 | Dorsolateral prefrontal cortex |
|  |  |  |  | 0.34 | 9 | Dorsolateral prefrontal cortex |
|  |  |  |  | 0.01 | 10 | Frontopolar area |
| 16 | -55 | 25 | 27 | 0.39 | 46 | Dorsolateral prefrontal cortex |
|  |  |  |  | 0.32 | 9 | Dorsolateral prefrontal cortex |
|  |  |  |  | 0.29 | 45 | pars triangularis Broca's area |
|  |  |  |  | 0 | 44 | pars opercularis |
| 17 | -43 | 26 | 47 | 0.82 | 8 | Includes Frontal eye fields |
|  |  |  |  | 0.18 | 9 | Dorsolateral prefrontal cortex |
| 18 | -22 | 29 | 59 | 0.6 | 8 | Includes Frontal eye fields |
|  |  |  |  | 0.4 | 6 | Pre-Motor and Supplementary Motor Cortex |
| 19 | -1 | 33 | 60 | 0.52 | 8 | Includes Frontal eye fields |
|  |  |  |  | 0.48 | 6 | Pre-Motor and Supplementary Motor Cortex |
| 20 | 22 | 32 | 59 | 0.6 | 8 | Includes Frontal eye fields |
|  |  |  |  | 0.4 | 6 | Pre-Motor and Supplementary Motor Cortex |
| 21 | 43 | 29 | 49 | 0.87 | 8 | Includes Frontal eye fields |
|  |  |  |  | 0.13 | 9 | Dorsolateral prefrontal cortex |
| 22 | 56 | 29 | 30 | 0.54 | 46 | Dorsolateral prefrontal cortex |
|  |  |  |  | 0.35 | 9 | Dorsolateral prefrontal cortex |
|  |  |  |  | 0.11 | 45 | pars triangularis Broca's area |
| 23 | -62 | 8 | 5 | 0.5 | 22 | Superior Temporal Gyrus |
|  |  |  |  | 0.3 | 44 | pars opercularis |
|  |  |  |  | 0.17 | 6 | Pre-Motor and Supplementary Motor Cortex |
|  |  |  |  | 0.03 | 45 | pars triangularis Broca's area |
| 24 | -60 | 13 | 26 | 0.51 | 9 | Dorsolateral prefrontal cortex |
|  |  |  |  | 0.22 | 45 | pars triangularis Broca's area |
|  |  |  |  | 0.2 | 44 | pars opercularis |
|  |  |  |  | 0.07 | 6 | Pre-Motor and Supplementary Motor Cortex |
| 25 | -49 | 17 | 47 | 0.64 | 8 | Includes Frontal eye fields |
|  |  |  |  | 0.2 | 9 | Dorsolateral prefrontal cortex |
|  |  |  |  | 0.15 | 6 | Pre-Motor and Supplementary Motor Cortex |
| 26 | -35 | 19 | 61 | 0.52 | 8 | Includes Frontal eye fields |
|  |  |  |  | 0.48 | 6 | Pre-Motor and Supplementary Motor Cortex |
| 27 | -14 | 22 | 67 | 1 | 6 | PreMotor and Supplementary Motor Cortex |
| 28 | 13 | 23 | 67 | 1 | 6 | PreMotor and Supplementary Motor Cortex |
| 29 | 34 | 21 | 61 | 0.52 | 6 | PreMotor and Supplementary Motor Cortex |
|  |  |  |  | 0.48 | 8 | Includes Frontal eye fields |
| 30 | 50 | 19 | 49 | 0.74 | 8 | Includes Frontal eye fields |
|  |  |  |  | 0.14 | 9 | Dorsolateral prefrontal cortex |
|  |  |  |  | 0.12 | 6 | Pre-Motor and Supplementary Motor Cortex |
| 31 | 62 | 17 | 28 | 0.6 | 9 | Dorsolateral prefrontal cortex |
|  |  |  |  | 0.27 | 45 | pars triangularis Broca's area |
|  |  |  |  | 0.08 | 44 | pars opercularis |
|  |  |  |  | 0.04 | 46 | Dorsolateral prefrontal cortex |
| 32 | 62 | 13 | 8 | 0.43 | 44 | pars opercularis; part of Broca's area |
|  |  |  |  | 0.23 | 45 | pars triangularis Broca's area |
|  |  |  |  | 0.22 | 22 | Superior Temporal Gyrus |
|  |  |  |  | 0.08 | 6 | Pre-Motor and Supplementary Motor Cortex |
|  |  |  |  | 0.04 | 47 | Inferior prefrontal gyrus |
| 33 | -68 | -7 | -11 | 1 | 21 | Middle Temporal gyrus |
| 34 | -65 | -1 | 20 | 0.58 | 6 | PreMotor and Supplementary Motor Cortex |
|  |  |  |  | 0.17 | 43 | Subcentral area |
|  |  |  |  | 0.08 | 22 | Superior Temporal Gyrus |
|  |  |  |  | 0.08 | 4 | Primary Motor Cortex |
|  |  |  |  | 0.06 | 44 | pars opercularis |
|  |  |  |  | 0.03 | 9 | Dorsolateral prefrontal cortex |
|  |  |  |  | 0.01 | 45 | pars triangularis Broca's area |
| 35 | -59 | 2 | 41 | 0.86 | 6 | PreMotor and Supplementary Motor Cortex |
|  |  |  |  | 0.11 | 9 | Dorsolateral prefrontal cortex |
|  |  |  |  | 0.03 | 8 | Includes Frontal eye fields |
| 36 | -44 | 9 | 59 | 0.87 | 6 | PreMotor and Supplementary Motor Cortex |
|  |  |  |  | 0.13 | 8 | Includes Frontal eye fields |
| 37 | -24 | 10 | 69 | 1 | 6 | PreMotor and Supplementary Motor Cortex |
| 38 | -2 | 11 | 71 | 1 | 6 | PreMotor and Supplementary Motor Cortex |
| 39 | 23 | 11 | 70 | 1 | 6 | PreMotor and Supplementary Motor Cortex |
| 40 | 45 | 9 | 59 | 0.82 | 6 | PreMotor and Supplementary Motor Cortex |
|  |  |  |  | 0.18 | 8 | Includes Frontal eye fields |
| 41 | 60 | 5 | 43 | 0.68 | 6 | PreMotor and Supplementary Motor Cortex |
|  |  |  |  | 0.21 | 9 | Dorsolateral prefrontal cortex |
|  |  |  |  | 0.11 | 8 | Includes Frontal eye fields |
| 42 | 67 | 4 | 22 | 0.62 | 6 | PreMotor and Supplementary Motor Cortex |
|  |  |  |  | 0.13 | 9 | Dorsolateral prefrontal cortex |
|  |  |  |  | 0.1 | 44 | pars opercularis |
|  |  |  |  | 0.07 | 45 | pars triangularis Broca's area |
|  |  |  |  | 0.06 | 4 | Primary Motor Cortex |
|  |  |  |  | 0.02 | 43 | Subcentral area |
| 43 | 68 | -2 | -8 | 0.85 | 21 | Middle Temporal gyrus |
|  |  |  |  | 0.15 | 22 | Superior Temporal Gyrus |
| 44 | -68 | -12 | 12 | 0.35 | 42 | Primary and Auditory Association Cortex |
|  |  |  |  | 0.33 | 43 | Subcentral area |
|  |  |  |  | 0.3 | 22 | Superior Temporal Gyrus |
|  |  |  |  | 0.02 | 40 | Supramarginal gyrus part of Wernicke's area |
|  |  |  |  | 0 | 4 | Primary Motor Cortex |
| 45 | -65 | -10 | 35 | 0.64 | 6 | PreMotor and Supplementary Motor Cortex |
|  |  |  |  | 0.15 | 3 | Primary Somatosensory Cortex |
|  |  |  |  | 0.11 | 4 | Primary Motor Cortex |
|  |  |  |  | 0.09 | 1 | Primary Somatosensory Cortex |
|  |  |  |  | 0.01 | 2 | Primary Somatosensory Cortex |
| 46 | -54 | -8 | 55 | 0.58 | 6 | PreMotor and Supplementary Motor Cortex |
|  |  |  |  | 0.27 | 3 | Primary Somatosensory Cortex |
|  |  |  |  | 0.14 | 4 | Primary Motor Cortex |
|  |  |  |  | 0.01 | 1 | Primary Somatosensory Cortex |
| 47 | -35 | -4 | 67 | 1 | 6 | PreMotor and Supplementary Motor Cortex |
| 48 | -14 | -2 | 75 | 1 | 6 | PreMotor and Supplementary Motor Cortex |
| 49 | 13 | -1 | 75 | 1 | 6 | PreMotor and Supplementary Motor Cortex |
| 50 | 34 | -3 | 68 | 1 | 6 | PreMotor and Supplementary Motor Cortex |
| 51 | 54 | -5 | 55 | 0.72 | 6 | PreMotor and Supplementary Motor Cortex |
|  |  |  |  | 0.14 | 3 | Primary Somatosensory Cortex |
|  |  |  |  | 0.13 | 4 | Primary Motor Cortex |
| 52 | 67 | -6 | 37 | 0.96 | 6 | PreMotor and Supplementary Motor Cortex |
|  |  |  |  | 0.03 | 4 | Primary Motor Cortex |
|  |  |  |  | 0.01 | 3 | Primary Somatosensory Cortex |
| 53 | 70 | -9 | 15 | 0.38 | 43 | Subcentral area |
|  |  |  |  | 0.25 | 22 | Superior Temporal Gyrus |
|  |  |  |  | 0.22 | 42 | Primary and Auditory Association Cortex |
|  |  |  |  | 0.08 | 6 | Pre-Motor and Supplementary Motor Cortex |
|  |  |  |  | 0.07 | 4 | Primary Motor Cortex |
|  |  |  |  | 0 | 40 | Supramarginal gyrus part of Wernicke's area |
| 54 | -71 | -26 | -3 | 0.7 | 21 | Middle Temporal gyrus |
|  |  |  |  | 0.22 | 22 | Superior Temporal Gyrus |
|  |  |  |  | 0.08 | 42 | Primary and Auditory Association Cortex |
| 55 | -68 | -22 | 28 | 0.35 | 40 | Supramarginal gyrus part of Wernicke's area |
|  |  |  |  | 0.24 | 2 | Primary Somatosensory Cortex |
|  |  |  |  | 0.18 | 1 | Primary Somatosensory Cortex |
|  |  |  |  | 0.11 | 43 | Subcentral area |
|  |  |  |  | 0.08 | 3 | Primary Somatosensory Cortex |
|  |  |  |  | 0.02 | 6 | Pre-Motor and Supplementary Motor Cortex |
|  |  |  |  | 0.02 | 42 | Primary and Auditory Association Cortex |
| 56 | -62 | -22 | 48 | 0.33 | 2 | Primary Somatosensory Cortex |
|  |  |  |  | 0.25 | 1 | Primary Somatosensory Cortex |
|  |  |  |  | 0.19 | 6 | Pre-Motor and Supplementary Motor Cortex |
|  |  |  |  | 0.14 | 3 | Primary Somatosensory Cortex |
|  |  |  |  | 0.08 | 4 | Primary Motor Cortex |
| 57 | -46 | -19 | 65 | 0.47 | 3 | Primary Somatosensory Cortex |
|  |  |  |  | 0.25 | 6 | Pre-Motor and Supplementary Motor Cortex |
|  |  |  |  | 0.15 | 4 | Primary Motor Cortex |
|  |  |  |  | 0.13 | 1 | Primary Somatosensory Cortex |
|  |  |  |  | 0 | 2 | Primary Somatosensory Cortex |
| 58 | -24 | -15 | 75 | 0.97 | 6 | PreMotor and Supplementary Motor Cortex |
|  |  |  |  | 0.03 | 4 | Primary Motor Cortex |
| 59 | -2 | -12 | 75 | 1 | 6 | PreMotor and Supplementary Motor Cortex |
| 60 | 24 | -13 | 75 | 1 | 6 | PreMotor and Supplementary Motor Cortex |
| 61 | 46 | -16 | 65 | 0.46 | 6 | PreMotor and Supplementary Motor Cortex |
|  |  |  |  | 0.32 | 3 | Primary Somatosensory Cortex |
|  |  |  |  | 0.19 | 4 | Primary Motor Cortex |
|  |  |  |  | 0.02 | 1 | Primary Somatosensory Cortex |
| 62 | 63 | -20 | 49 | 0.26 | 1 | Primary Somatosensory Cortex |
|  |  |  |  | 0.22 | 3 | Primary Somatosensory Cortex |
|  |  |  |  | 0.22 | 2 | Primary Somatosensory Cortex |
|  |  |  |  | 0.21 | 6 | Pre-Motor and Supplementary Motor Cortex |
|  |  |  |  | 0.09 | 4 | Primary Motor Cortex |
|  |  |  |  | 0.01 | 40 | Supramarginal gyrus part of Wernicke's area |
| 63 | 70 | -20 | 30 | 0.26 | 40 | Supramarginal gyrus part of Wernicke's area |
|  |  |  |  | 0.21 | 2 | Primary Somatosensory Cortex |
|  |  |  |  | 0.2 | 1 | Primary Somatosensory Cortex |
|  |  |  |  | 0.16 | 3 | Primary Somatosensory Cortex |
|  |  |  |  | 0.07 | 6 | Pre-Motor and Supplementary Motor Cortex |
|  |  |  |  | 0.07 | 43 | Subcentral area |
|  |  |  |  | 0.01 | 4 | Primary Motor Cortex |
| 64 | 73 | -22 | 0 | 0.42 | 21 | Middle Temporal gyrus |
|  |  |  |  | 0.4 | 22 | Superior Temporal Gyrus |
|  |  |  |  | 0.18 | 42 | Primary and Auditory Association Cortex |
| 65 | -69 | -36 | 14 | 0.75 | 22 | Superior Temporal Gyrus |
|  |  |  |  | 0.21 | 42 | Primary and Auditory Association Cortex |
|  |  |  |  | 0.04 | 40 | Supramarginal gyrus part of Wernicke's area |
| 66 | -66 | -33 | 40 | 0.78 | 40 | Supramarginal gyrus part of Wernicke's area |
|  |  |  |  | 0.16 | 2 | Primary Somatosensory Cortex |
|  |  |  |  | 0.06 | 1 | Primary Somatosensory Cortex |
| 67 | -54 | -29 | 57 | 0.41 | 2 | Primary Somatosensory Cortex |
|  |  |  |  | 0.37 | 40 | Supramarginal gyrus part of Wernicke's area |
|  |  |  |  | 0.2 | 1 | Primary Somatosensory Cortex |
|  |  |  |  | 0.02 | 3 | Primary Somatosensory Cortex |
| 68 | -37 | -26 | 72 | 0.33 | 6 | PreMotor and Supplementary Motor Cortex |
|  |  |  |  | 0.3 | 3 | Primary Somatosensory Cortex |
|  |  |  |  | 0.3 | 4 | Primary Motor Cortex |
|  |  |  |  | 0.07 | 1 | Primary Somatosensory Cortex |
|  |  |  |  | 0.01 | 2 | Primary Somatosensory Cortex |
| 69 | -14 | -25 | 79 | 0.64 | 6 | PreMotor and Supplementary Motor Cortex |
|  |  |  |  | 0.34 | 4 | Primary Motor Cortex |
|  |  |  |  | 0.02 | 3 | Primary Somatosensory Cortex |
| 70 | 14 | -25 | 79 | 0.72 | 6 | PreMotor and Supplementary Motor Cortex |
|  |  |  |  | 0.28 | 4 | Primary Motor Cortex |
|  |  |  |  | 0 | 3 | Primary Somatosensory Cortex |
| 71 | 36 | -25 | 73 | 0.43 | 4 | Primary Motor Cortex |
|  |  |  |  | 0.38 | 6 | Pre-Motor and Supplementary Motor Cortex |
|  |  |  |  | 0.18 | 3 | Primary Somatosensory Cortex |
|  |  |  |  | 0.02 | 1 | Primary Somatosensory Cortex |
| 72 | 55 | -29 | 59 | 0.35 | 40 | Supramarginal gyrus part of Wernicke's area |
|  |  |  |  | 0.3 | 2 | Primary Somatosensory Cortex |
|  |  |  |  | 0.24 | 1 | Primary Somatosensory Cortex |
|  |  |  |  | 0.1 | 3 | Primary Somatosensory Cortex |
| 73 | 68 | -31 | 42 | 0.62 | 40 | Supramarginal gyrus part of Wernicke's area |
|  |  |  |  | 0.19 | 1 | Primary Somatosensory Cortex |
|  |  |  |  | 0.16 | 2 | Primary Somatosensory Cortex |
|  |  |  |  | 0.02 | 3 | Primary Somatosensory Cortex |
|  |  |  |  | 0.01 | 4 | Primary Motor Cortex |
| 74 | 71 | -32 | 16 | 0.42 | 22 | Superior Temporal Gyrus |
|  |  |  |  | 0.36 | 42 | Primary and Auditory Association Cortex |
|  |  |  |  | 0.22 | 40 | Supramarginal gyrus part of Wernicke's area |
| 75 | -69 | -47 | -3 | 0.76 | 21 | Middle Temporal gyrus |
|  |  |  |  | 0.19 | 22 | Superior Temporal Gyrus |
|  |  |  |  | 0.05 | 37 | Fusiform gyrus |
| 76 | -67 | -45 | 26 | 0.76 | 40 | Supramarginal gyrus part of Wernicke's area |
|  |  |  |  | 0.24 | 22 | Superior Temporal Gyrus |
| 77 | -61 | -43 | 47 | 1 | 40 | Supramarginal gyrus part of Wernicke's area |
| 78 | -45 | -38 | 65 | 0.37 | 40 | Supramarginal gyrus part of Wernicke's area |
|  |  |  |  | 0.32 | 2 | Primary Somatosensory Cortex |
|  |  |  |  | 0.18 | 1 | Primary Somatosensory Cortex |
|  |  |  |  | 0.11 | 5 | Somatosensory Association Cortex |
|  |  |  |  | 0.03 | 3 | Primary Somatosensory Cortex |
| 79 | -23 | -39 | 75 | 0.4 | 3 | Primary Somatosensory Cortex |
|  |  |  |  | 0.3 | 5 | Somatosensory Association Cortex |
|  |  |  |  | 0.16 | 2 | Primary Somatosensory Cortex |
|  |  |  |  | 0.1 | 4 | Primary Motor Cortex |
|  |  |  |  | 0.03 | 1 | Primary Somatosensory Cortex |
| 80 | 24 | -37 | 77 | 0.41 | 3 | Primary Somatosensory Cortex |
|  |  |  |  | 0.28 | 4 | Primary Motor Cortex |
|  |  |  |  | 0.16 | 5 | Somatosensory Association Cortex |
|  |  |  |  | 0.13 | 2 | Primary Somatosensory Cortex |
|  |  |  |  | 0.02 | 1 | Primary Somatosensory Cortex |
| 81 | 46 | -38 | 65 | 0.3 | 2 | Primary Somatosensory Cortex |
|  |  |  |  | 0.3 | 40 | Supramarginal gyrus part of Wernicke's area |
|  |  |  |  | 0.17 | 1 | Primary Somatosensory Cortex |
|  |  |  |  | 0.13 | 5 | Somatosensory Association Cortex |
|  |  |  |  | 0.1 | 3 | Primary Somatosensory Cortex |
| 82 | 63 | -42 | 48 | 1 | 40 | Supramarginal gyrus part of Wernicke's area |
| 83 | 69 | -43 | 26 | 0.75 | 40 | Supramarginal gyrus part of Wernicke's area |
|  |  |  |  | 0.24 | 22 | Superior Temporal Gyrus |
|  |  |  |  | 0.01 | 42 | Primary and Auditory Association Cortex |
| 84 | 71 | -44 | -1 | 0.68 | 21 | Middle Temporal gyrus |
|  |  |  |  | 0.28 | 22 | Superior Temporal Gyrus |
|  |  |  |  | 0.04 | 37 | Fusiform gyrus |
| 85 | -66 | -56 | 8 | 0.56 | 21 | Middle Temporal gyrus |
|  |  |  |  | 0.38 | 22 | Superior Temporal Gyrus |
|  |  |  |  | 0.03 | 39 | Angular gyrus |
|  |  |  |  | 0.03 | 37 | Fusiform gyrus |
| 86 | -62 | -55 | 35 | 0.83 | 40 | Supramarginal gyrus part of Wernicke's area |
|  |  |  |  | 0.17 | 39 | Angular gyrus |
| 87 | -53 | -53 | 53 | 1 | 40 | Supramarginal gyrus part of Wernicke's area |
| 88 | -35 | -52 | 70 | 0.49 | 5 | Somatosensory Association Cortex |
|  |  |  |  | 0.41 | 7 | Somatosensory Association Cortex |
|  |  |  |  | 0.08 | 40 | Supramarginal gyrus part of Wernicke's area |
|  |  |  |  | 0.02 | 2 | Primary Somatosensory Cortex |
| 89 | 35 | -51 | 70 | 0.49 | 5 | Somatosensory Association Cortex |
|  |  |  |  | 0.46 | 7 | Somatosensory Association Cortex |
|  |  |  |  | 0.03 | 2 | Primary Somatosensory Cortex |
|  |  |  |  | 0.02 | 40 | Supramarginal gyrus part of Wernicke's area |
| 90 | 55 | -53 | 54 | 1 | 40 | Supramarginal gyrus part of Wernicke's area |
| 91 | 63 | -55 | 34 | 0.85 | 40 | Supramarginal gyrus part of Wernicke's area |
|  |  |  |  | 0.15 | 39 | Angular gyrus |
| 92 | 66 | -55 | 8 | 0.51 | 21 | Middle Temporal gyrus |
|  |  |  |  | 0.4 | 22 | Superior Temporal Gyrus |
|  |  |  |  | 0.05 | 39 | Angular gyrus |
|  |  |  |  | 0.04 | 37 | Fusiform gyrus |
| 93 | -60 | -67 | -6 | 0.61 | 37 | Fusiform gyrus |
|  |  |  |  | 0.33 | 19 | V3 |
|  |  |  |  | 0.06 | 21 | Middle Temporal gyrus |
| 94 | -57 | -64 | 38 | 0.56 | 39 | Angular gyrus; part of Wernicke's area |
|  |  |  |  | 0.44 | 40 | Supramarginal gyrus part of Wernicke's area |
| 95 | -45 | -64 | 54 | 0.54 | 40 | Supramarginal gyrus part of Wernicke's area |
|  |  |  |  | 0.44 | 7 | Somatosensory Association Cortex |
|  |  |  |  | 0.02 | 39 | Angular gyrus |
| 96 | -26 | -62 | 70 | 0.99 | 7 | Somatosensory Association Cortex |
|  |  |  |  | 0.01 | 5 | Somatosensory Association Cortex |
| 97 | 26 | -59 | 72 | 0.96 | 7 | Somatosensory Association Cortex |
|  |  |  |  | 0.04 | 5 | Somatosensory Association Cortex |
| 98 | 46 | -61 | 57 | 0.51 | 7 | Somatosensory Association Cortex |
|  |  |  |  | 0.49 | 40 | Supramarginal gyrus part of Wernicke's area |
| 99 | 57 | -65 | 38 | 0.67 | 39 | Angular gyrus; part of Wernicke's area |
|  |  |  |  | 0.33 | 40 | Supramarginal gyrus part of Wernicke's area |
| 100 | 59 | -68 | -7 | 0.5 | 37 | Fusiform gyrus |
|  |  |  |  | 0.49 | 19 | V3 |
|  |  |  |  | 0.01 | 21 | Middle Temporal gyrus |
| 101 | -50 | -75 | 37 | 0.9 | 39 | Angular gyrus; part of Wernicke's area |
|  |  |  |  | 0.1 | 19 | V3 |
| 102 | -36 | -73 | 54 | 0.96 | 7 | Somatosensory Association Cortex |
|  |  |  |  | 0.04 | 19 | V3 |
| 103 | -15 | -72 | 65 | 1 | 7 | Somatosensory Association Cortex |
| 104 | 16 | -72 | 67 | 1 | 7 | Somatosensory Association Cortex |
| 105 | 34 | -71 | 55 | 1 | 7 | Somatosensory Association Cortex |
| 106 | 49 | -74 | 39 | 0.64 | 39 | Angular gyrus; part of Wernicke's area |
|  |  |  |  | 0.3 | 19 | V3 |
|  |  |  |  | 0.05 | 7 | Somatosensory Association Cortex |
|  |  |  |  | 0 | 40 | Supramarginal gyrus part of Wernicke's area |
| 107 | -25 | -81 | 51 | 0.82 | 7 | Somatosensory Association Cortex |
|  |  |  |  | 0.18 | 19 | V3 |
| 108 | 2 | -78 | 56 | 1 | 7 | Somatosensory Association Cortex |
| 109 | 26 | -79 | 52 | 0.94 | 7 | Somatosensory Association Cortex |
|  |  |  |  | 0.06 | 19 | V3 |
| 110 | -14 | -89 | 43 | 0.78 | 19 | V3 |
|  |  |  |  | 0.22 | 7 | Somatosensory Association Cortex |
| 111 | 17 | -88 | 43 | 0.75 | 19 | V3 |
|  |  |  |  | 0.25 | 7 | Somatosensory Association Cortex |
| 112 | -24 | -93 | 28 | 0.97 | 19 | V3 |
|  |  |  |  | 0.03 | 18 | Visual Association Cortex (V2) |
| 113 | 1 | -95 | 32 | 0.99 | 19 | V3 |
|  |  |  |  | 0.01 | 18 | Visual Association Cortex (V2) |
| 114 | 26 | -92 | 30 | 0.99 | 19 | V3 |
|  |  |  |  | 0.01 | 18 | Visual Association Cortex (V2) |
| 115 | -35 | -95 | 12 | 0.57 | 19 | V3 |
|  |  |  |  | 0.43 | 18 | Visual Association Cortex (V2) |
| 116 | -14 | -102 | 18 | 0.74 | 18 | Visual Association Cortex (V2) |
|  |  |  |  | 0.26 | 19 | V3 |
| 117 | 17 | -101 | 19 | 0.62 | 18 | Visual Association Cortex (V2) |
|  |  |  |  | 0.38 | 19 | V3 |
| 118 | 34 | -94 | 13 | 0.67 | 19 | V3 |
|  |  |  |  | 0.33 | 18 | Visual Association Cortex (V2) |
| 119 | -23 | -102 | 6 | 0.85 | 18 | Visual Association Cortex (V2) |
|  |  |  |  | 0.15 | 19 | V3 |
| 120 | -1 | -102 | 11 | 1 | 18 | Visual Association Cortex (V2) |
| 121 | 24 | -100 | 7 | 0.8 | 18 | Visual Association Cortex (V2) |
|  |  |  |  | 0.2 | 19 | V3 |
| 122 | -15 | -105 | -1 | 0.95 | 18 | Visual Association Cortex (V2) |
|  |  |  |  | 0.05 | 17 | Primary Visual Cortex (V1) |
| 123 | 16 | -103 | 1 | 0.97 | 18 | Visual Association Cortex (V2) |
|  |  |  |  | 0.03 | 17 | Primary Visual Cortex (V1) |
| 124 | -24 | -100 | -11 | 0.76 | 18 | Visual Association Cortex (V2) |
|  |  |  |  | 0.24 | 17 | Primary Visual Cortex (V1) |
| 125 | -5 | -103 | -5 | 0.8 | 18 | Visual Association Cortex (V2) |
|  |  |  |  | 0.2 | 17 | Primary Visual Cortex (V1) |
| 126 | 23 | -99 | -12 | 0.66 | 18 | Visual Association Cortex (V2) |
|  |  |  |  | 0.34 | 17 | Primary Visual Cortex (V1) |
| 127 | -17 | -99 | -18 | 0.56 | 17 | Primary Visual Cortex (V1) |
|  |  |  |  | 0.44 | 18 | Visual Association Cortex (V2) |
| 128 | 15 | -97 | -17 | 0.53 | 17 | Primary Visual Cortex (V1) |
|  |  |  |  | 0.47 | 18 | Visual Association Cortex (V2) |
| 129 | -50 | 44 | 12 | 0.79 | 46 | Dorsolateral prefrontal cortex |
|  |  |  |  | 0.12 | 10 | Frontopolar area |
|  |  |  |  | 0.09 | 45 | pars triangularis Broca's area |
| 130 | 51 | 48 | 15 | 0.72 | 46 | Dorsolateral prefrontal cortex |
|  |  |  |  | 0.27 | 10 | Frontopolar area |
|  |  |  |  | 0 | 45 | pars triangularis Broca's area |
| 131 | -48 | -84 | 1 | 0.77 | 19 | V3 |
|  |  |  |  | 0.19 | 18 | Visual Association Cortex (V2) |
|  |  |  |  | 0.04 | 37 | Fusiform gyrus |
| 132 | 48 | -85 | 0 | 0.72 | 19 | V3 |
|  |  |  |  | 0.28 | 18 | Visual Association Cortex (V2) |
| 133 | -49 | -82 | 19 | 0.67 | 19 | V3 |
|  |  |  |  | 0.33 | 39 | Angular gyrus |
| 134 | 48 | -82 | 19 | 0.82 | 19 | V3 |
|  |  |  |  | 0.18 | 39 | Angular gyrus |
|  |  |  |  | 0.01 | 18 | Visual Association Cortex (V2) |

Supplementary Table 2. Whole-brain results for anticipated > unanticipated (anticipation phase) (stutterers only) (deoxyhemoglobin [HbR]). Regions on first line indicate locations of peak within cluster; indented lines represent other regions within cluster (Prob = probability, or the relative contributions of each region within the cluster). Positive t-values indicate anticipated > unanticipated; negative t-values indicate anticipated < unanticipated. Hemi = hemisphere; MNI = Montreal Neurological Institute.

| Region | Brodmann Area | Hemi | Peak MNI | | | Voxels | | t | p | Prob |
| --- | --- | --- | --- | --- | --- | --- | --- | --- | --- | --- |
|  |  |  | x | y | z | |  |  |  |  |
| Supramarginal Gyrus |  | L | -62 | -44 | 44 | | 935 | 2.70 | .0067 |  |
| Supramarginal Gyrus | 40 | L |  |  |  | |  |  |  | .97 |
| Visual Association Cortex (V3) |  | L | -44 | -86 | 8 | | 272 | -3.04 | .0031 |  |
| Visual Association Cortex (V3) | 19 | L |  |  |  | |  |  |  | .74 |
| Visual Association Cortex (V2) | 18 | L |  |  |  | |  |  |  | .24 |
| Visual Association Cortex (V2) |  | R | 40 | -74 | 28 | | 246 | -2.22 | .0189 |  |
| Visual Association Cortex (V2) | 18 | R |  |  |  | |  |  |  | .58 |
| Visual Association Cortex (V3) | 19 | R |  |  |  | |  |  |  | .42 |
| Visual Association Cortex (V3) |  | R | 40 | -90 | 14 | | 245 | 3.67 | .0007 |  |
| Visual Association Cortex (V3) | 19 | R |  |  |  | |  |  |  | .81 |
| Visual Association Cortex (V2) | 18 | R |  |  |  | |  |  |  | .18 |
| Somatosensory Association Cortex |  | L | -8 | -80 | 52 | | 220 | -2.40 | .0128 |  |
| Somatosensory Association Cortex | 7 | L |  |  |  | |  |  |  | .84 |
| Visual Association Cortex (V3) | 19 | L |  |  |  | |  |  |  | .16 |
| Premotor/Supplementary Motor |  | R | 14 | -6 | 70 | | 215 | -2.28 | .0167 |  |
| Premotor/Supplementary Motor | 6 | R |  |  |  | |  |  |  | 1.0 |
| Dorsolateral Prefrontal Cortex |  | R | 52 | 20 | 40 | | 174 | 2.97 | .0037 |  |
| Dorsolateral Prefrontal Cortex | 9 | R |  |  |  | |  |  |  | .54 |
| Frontal Eye Fields | 8 | R |  |  |  | |  |  |  | .34 |
| Frontopolar area |  | L | -20 | 48 | 24 | | 128 | -2.44 | .0117 |  |
| Frontopolar area | 10 | L |  |  |  | |  |  |  | .60 |
| Dorsolateral Prefrontal Cortex | 9 | L |  |  |  | |  |  |  | .40 |
| Inferior prefrontal gyrus |  | L | -46 | 30 | 4 | | 84 | -2.16 | .0214 |  |
| Inferior prefrontal gyrus | 47 | L |  |  |  | |  |  |  | .48 |
| Pars Triangularis (Broca’s area) | 45 | L |  |  |  | |  |  |  | .34 |
| Dorsolateral Prefrontal Cortex | 46 | L |  |  |  | |  |  |  | .16 |
| Premotor/Supplementary Motor |  | R | 54 | -12 | 54 | | 69 | 2.83 | .0050 |  |
| Premotor/Supplementary Motor | 6 | R |  |  |  | |  |  |  | .50 |
| Primary Somatosensory Cortex | 3 | R |  |  |  | |  |  |  | .23 |
| Primary Motor Cortex | 4 | R |  |  |  | |  |  |  | .11 |
| Primary Somatosensory Cortex | 1 | R |  |  |  | |  |  |  | .10 |
| Premotor/Supplementary Motor |  | L | -42 | 8 | 60 | | 66 | 2.75 | .0059 |  |
| Premotor/Supplementary Motor | 6 | L |  |  |  | |  |  |  | .82 |
| Frontal Eye Fields | 8 | L |  |  |  | |  |  |  | .18 |
| Superior Temporal Gyrus |  | R | 68 | -40 | 4 | | 63 | 2.64 | .0077 |  |
| Superior Temporal Gyrus |  | R |  |  |  | |  |  |  | .53 |
| Middle Temporal Gyrus |  | R |  |  |  | |  |  |  | .42 |
| Supramarginal Gyrus |  | R | 56 | -36 | 52 | | 61 | 2.70 | .0067 |  |
| Supramarginal Gyrus | 40 | R |  |  |  | |  |  |  | .72 |
| Primary Somatosensory Cortex | 2 | R |  |  |  | |  |  |  | .20 |

Supplementary Table 3. Whole-brain results for anticipated > unanticipated (anticipation phase) (stutterers only) (oxyhemoglobin [HbO). Regions on first line indicate locations of peak within cluster; indented lines represent other regions within cluster (Prob = probability, or the relative contributions of each region within the cluster). Positive t-values indicate anticipated > unanticipated; negative t-values indicate anticipated < unanticipated. Hemi = hemisphere.

| Region | Brodmann Area | Hemi | Peak MNI | | | Voxels | | t | p | Prob |
| --- | --- | --- | --- | --- | --- | --- | --- | --- | --- | --- |
|  |  |  | x | y | z | |  |  |  |  |
| Dorsolateral Prefrontal Cortex |  | L | -40 | 20 | 38 | | 2528 | -3.99 | .0003 |  |
| Dorsolateral Prefrontal Cortex | 9 | L |  |  |  | |  |  |  | .52 |
| Frontal Eye Fields | 8 | L |  |  |  | |  |  |  | .46 |
| Dorsolateral Prefrontal Cortex |  | R | 44 | 44 | 20 | | 410 | -3.62 | .0008 |  |
| Dorsolateral Prefrontal Cortex | 46 | R |  |  |  | |  |  |  | .63 |
| Frontopolar area | 10 | R |  |  |  | |  |  |  | .34 |
| Premotor/Supplementary Motor |  | L | -36 | 6 | 60 | | 265 | 4.10 | .0003 |  |
| Premotor/Supplementary Motor | 6 | L |  |  |  | |  |  |  | .90 |
| Fusiform gyrus |  | R | 60 | -52 | -16 | | 121 | 2.26 | .0174 |  |
| Fusiform Gyrus |  | R |  |  |  | |  |  |  | .61 |
| Inferior Temporal |  | R |  |  |  | |  |  |  | .28 |
| Middle Temporal Gyrus |  | R |  |  |  | |  |  |  | .10 |
| Premotor/Supplementary Motor |  | R | 62 | 2 | 38 | | 120 | -2.97 | .0037 |  |
| Premotor/Supplementary Motor | 6 | R |  |  |  | |  |  |  | .72 |
| Dorsolateral Prefrontal Cortex | 9 | R |  |  |  | |  |  |  | .24 |
| Dorsolateral Prefrontal Cortex |  | L | -22 | 52 | 38 | | 105 | 2.91 | .0042 |  |
| Dorsolateral Prefrontal Cortex | 9 | L |  |  |  | |  |  |  | .63 |
| Frontal Eye Fields | 8 | L |  |  |  | |  |  |  | .20 |
| Frontopolar area | 10 | L |  |  |  | |  |  |  | .17 |
| Premotor/Supplementary Motor |  | R | 6 | 32 | 62 | | 95 | 3.18 | .0023 |  |
| Premotor/Supplementary Motor | 6 | R |  |  |  | |  |  |  | .56 |
| Frontal Eye Fields | 8 | R |  |  |  | |  |  |  | .44 |
| Visual Association Cortex (V3) |  | R | 50 | -66 | -12 | | 76 | -2.29 | .0161 |  |
| Visual Association Cortex (V3) | 19 | R |  |  |  | |  |  |  | .64 |
| Fusiform Gyrus | 37 | R |  |  |  | |  |  |  | .28 |
| Somatosensory Association Cortex |  | R | 4 | -84 | 50 | | 75 | 2.60 | .0084 |  |
| Somatosensory Association Cortex | 7 | R |  |  |  | |  |  |  | .68 |
| Visual Association Cortex (V3) | 19 | R |  |  |  | |  |  |  | .32 |
| Premotor/Supplementary Motor |  | L | -58 | -16 | 46 | | 74 | -2.36 | .0140 |  |
| Premotor/Supplementary Motor | 6 | L |  |  |  | |  |  |  | .41 |
| Primary Somatosensory Cortex | 3 | L |  |  |  | |  |  |  | .20 |
| Primary Somatosensory Cortex | 1 | L |  |  |  | |  |  |  | .16 |
| Primary Somatosensory Cortex | 2 | L |  |  |  | |  |  |  | .15 |
| Somatosensory Association Cortex |  | L | -22 | -52 | 68 | | 71 | 2.15 | .0216 |  |
| Somatosensory Association Cortex | 7 | L |  |  |  | |  |  |  | .66 |
| Somatosensory Association Cortex | 5 | L |  |  |  | |  |  |  | .31 |
| Visual Association Cortex (V2) |  | R | 12 | -94 | -14 | | 60 | -2.33 | .0149 |  |
| Visual Association Cortex (V2) | 18 | R |  |  |  | |  |  |  | .65 |
| Primary Visual Cortex | 17 | R |  |  |  | |  |  |  | .35 |
| Premotor/Supplementary Motor |  | L | -2 | -30 | 78 | | 57 | 2.88 | .0045 |  |
| Premotor/Supplementary Motor | 6 | L |  |  |  | |  |  |  | .65 |
| Primary Motor Cortex | 4 | L |  |  |  | |  |  |  | .26 |
| Visual Association Cortex (V2) |  | L | -24 | -88 | -22 | | 53 | 2.08 | .0249 |  |
| Visual Association Cortex (V2) | 18 | L |  |  |  | |  |  |  | .84 |
| Primary Visual Cortex | 17 | R |  |  |  | |  |  |  | .16 |

Supplementary Table 4. Whole-brain results for stutterers > controls analysis (anticipation phase) (deoxyhemoglobin [HbR]). Regions on first line indicate locations of peak within cluster; indented lines represent other regions within cluster (Prob = probability, or the relative contributions of each region within the cluster). Positive t-values indicate anticipated > unanticipated; negative t-values indicate anticipated < unanticipated. Hemi = hemisphere; MNI = Montreal Neurological Institute.

| Region | Brodmann Area | Hemi | Peak MNI | | | Voxels | | t | | p | | Prob | |  |
| --- | --- | --- | --- | --- | --- | --- | --- | --- | --- | --- | --- | --- | --- | --- |
|  |  |  | x | y | z | |  | |  | |  | |  | |
| Frontal Eye Fields |  | R | 26 | 22 | 54 | | 6,016 | | 3.41 | | .0007 | |  | |
| Frontal Eye Fields | 8 | R |  |  |  | |  | |  | |  | | .51 | |
| Premotor/Supplementary Motor | 6 | R |  |  |  | |  | |  | |  | | .49 | |
| V3 |  | R | 36 | -82 | 8 | | 1,074 | | -2.86 | | .0033 | |  | |
| Visual Association Cortex (V3) | 19 | R |  |  |  | |  | |  | |  | | .80 | |
| Visual Association Cortex (V2) | 18 | R |  |  |  | |  | |  | |  | | .20 | |
| Middle Temporal Gyrus |  | L | -64 | -18 | -10 | | 425 | | 2.57 | | .0069 | |  | |
| Middle Temporal Gyrus | 21 | L |  |  |  | |  | |  | |  | | .83 | |
| Somatosensory Association Cortex |  | L | -16 | -56 | 74 | | 396 | | 2.75 | | .0044 | |  | |
| Somatosensory Association Cortex | 7 | L |  |  |  | |  | |  | |  | | .72 | |
| Somatosensory Association Cortex | 5 | L |  |  |  | |  | |  | |  | | .24 | |
| Visual Association Cortex (V3) |  | R | 46 | -74 | -16 | | 124 | | -2.45 | | .0093 | |  | |
| Visual Association Cortex (V3) | 19 | R |  |  |  | |  | |  | |  | | .65 | |
| Visual Association Cortex (V2) | 18 | R |  |  |  | |  | |  | |  | | .35 | |
| Supramarginal Gyrus |  | L | -52 | -66 | 46 | | 106 | | -2.23 | | .0157 | |  | |
| Supramarginal Gyrus | 40 | L |  |  |  | |  | |  | |  | | .46 | |
| Angular Gyrus | 39 | L |  |  |  | |  | |  | |  | | .37 | |
| Somatosensory Association Cortex | 7 | L |  |  |  | |  | |  | |  | | .13 | |
| Middle Temporal Gyrus |  | L | -68 | -48 | -8 | | 103 | | 2.68 | | .0052 | |  | |
| Middle Temporal Gyrus | 21 | L |  |  |  | |  | |  | |  | | .58 | |
| Fusiform Gyrus | 37 | L |  |  |  | |  | |  | |  | | .23 | |
| Inferior Temporal Gyrus | 20 | L |  |  |  | |  | |  | |  | | .12 | |
| Somatosensory Association Cortex |  | R | 36 | -70 | 56 | | 70 | | 2.97 | | .0025 | |  | |
| Somatosensory Association Cortex | 7 | R |  |  |  | |  | |  | |  | | .94 | |

Supplementary Table 5. Whole-brain results for stutterers > controls analysis (anticipation phase) (oxyhemoglobin [HbO]). Regions on first line indicate locations of peak within cluster; indented lines represent other regions within cluster (Prob = probability, or the relative contributions of each region within the cluster). Positive t-values indicate anticipated > unanticipated; negative t-values indicate anticipated < unanticipated. Hemi = hemisphere; MNI = Montreal Neurological Institute.

| Region | Brodmann Area | Hemi | Peak MNI | | | Voxels | | t | | p | | Prob | |  |
| --- | --- | --- | --- | --- | --- | --- | --- | --- | --- | --- | --- | --- | --- | --- |
|  |  |  | x | y | z | |  | |  | |  | |  | |
| Somatosensory Association Cortex |  | R | 18 | -80 | 54 | | 1,729 | | 2.68 | | .0053 | |  | |
| Somatosensory Association Cortex | 7 | R |  |  |  | |  | |  | |  | | .87 | |
| Visual Association Cortex (V3) | 19 | R |  |  |  | |  | |  | |  | | .13 | |
| Premotor/Supplementary Motor |  | R | 60 | -2 | 42 | | 800 | | -3.35 | | .0009 | |  | |
| Premotor/Supplementary Motor | 6 | R |  |  |  | |  | |  | |  | | .81 | |
| Inferior Temporal gyrus |  | R | 60 | -32 | -20 | | 428 | | 2.13 | | .0194 | |  | |
| Inferior Temporal gyrus | 20 | R |  |  |  | |  | |  | |  | | .75 | |
| Middle Temporal gyrus | 21 | R |  |  |  | |  | |  | |  | | .24 | |
| Premotor/Supplementary Motor |  | L | -60 | -20 | 46 | | 390 | | -3.56 | | .0005 | |  | |
| Premotor/Supplementary Motor | 6 | L |  |  |  | |  | |  | |  | | .26 | |
| Primary Somatosensory Cortex | 2 | L |  |  |  | |  | |  | |  | | .23 | |
| Primary Somatosensory Cortex | 3 | L |  |  |  | |  | |  | |  | | .17 | |
| Primary Somatosensory Cortex | 1 | L |  |  |  | |  | |  | |  | | .16 | |
| Supramarginal Gyrus | 40 | L |  |  |  | |  | |  | |  | | .12 | |
| Frontopolar area |  | R | 42 | 56 | 14 | | 185 | | 3.00 | | .0022 | |  | |
| Frontopolar area | 10 | R |  |  |  | |  | |  | |  | | .82 | |
| Dorsolateral Prefrontal Cortex | 46 | R |  |  |  | |  | |  | |  | | .18 | |
| Fusiform Gyrus |  | L | -58 | -66 | -12 | | 147 | | -2.56 | | .0071 | |  | |
| Fusiform Gyrus | 37 | L |  |  |  | |  | |  | |  | | .48 | |
| Visual Association Cortex (V3) | 19 | L |  |  |  | |  | |  | |  | | .41 | |
| Visual Association Cortex (V2) |  | R | 14 | -94 | 10 | | 90 | | 2.35 | | .0117 | |  | |
| Visual Association Cortex (V2) | 18 | R |  |  |  | |  | |  | |  | | .91 | |
| Premotor/Supplementary Motor |  | L | -28 | 16 | 54 | | 88 | | 2.56 | | .0071 | |  | |
| Premotor/Supplementary Motor | 6 | L |  |  |  | |  | |  | |  | | .55 | |
| Frontal Eye Fields | 8 | L |  |  |  | |  | |  | |  | | .45 | |
| Angular Gyrus |  | L | -62 | -62 | 12 | | 84 | | -2.45 | | .0093 | |  | |
| Angular Gyrus | 39 | L |  |  |  | |  | |  | |  | | .26 | |
| Superior Temporal Gyrus | 22 | L |  |  |  | |  | |  | |  | | .25 | |
| Middle Temporal Gyrus | 21 | L |  |  |  | |  | |  | |  | | .24 | |
| Visual Association Cortex (V3) | 19 | L |  |  |  | |  | |  | |  | | .12 | |
| Fusiform Gyrus | 37 | L |  |  |  | |  | |  | |  | | .11 | |
| Dorsolateral Prefrontal Cortex |  | R | 22 | 52 | 32 | | 83 | | -2.10 | | .0211 | |  | |
| Dorsolateral Prefrontal Cortex | 9 | R |  |  |  | |  | |  | |  | | .64 | |
| Frontopolar area | 10 | R |  |  |  | |  | |  | |  | | .32 | |
| Visual Association Cortex (V2) |  | R | 20 | -102 | -4 | | 79 | | 2.22 | | .0158 | |  | |
| Visual Association Cortex (V2) | 18 | R |  |  |  | |  | |  | |  | | .85 | |
| Primary Visual Cortex (V1) | 17 | R |  |  |  | |  | |  | |  | | .15 | |
| Fusiform Gyrus |  | R | 60 | -62 | -14 | | 62 | | -2.53 | | .0075 | |  | |
| Fusiform Gyrus | 37 | R |  |  |  | |  | |  | |  | | .54 | |
| Visual Association Cortex (V3) | 19 | R |  |  |  | |  | |  | |  | | .30 | |
| Inferior Temporal gyrus | 20 | R |  |  |  | |  | |  | |  | | .13 | |

Supplementary Table 6. Whole-brain results for stuttered > fluent analysis (anticipation phase) (stutterers only) (deoxyhemoglobin [HbR]). Regions on first line indicate locations of peak within cluster; indented lines represent other regions within cluster (Prob = probability, or the relative contributions of each region within the cluster). Positive t-values indicate anticipated > unanticipated; negative t-values indicate anticipated < unanticipated. Hemi = hemisphere; MNI = Montreal Neurological Institute.

| Region | Brodmann Area | Hemi | Peak MNI | | | Voxels | t | p | Prob |
| --- | --- | --- | --- | --- | --- | --- | --- | --- | --- |
|  |  |  | x | y | z |  |  |  |  |
| Visual Association Cortex (V3) |  | L | -30 | -86 | 22 | 1,383 | 3.12 | .0026 |  |
| Visual Association Cortex (V3) | 19 | L |  |  |  |  |  |  | .99 |
| Frontal Eye Fields |  | R | 50 | 16 | 44 | 1,034 | 4.48 | .0001 |  |
| Frontal Eye Fields | 8 | R |  |  |  |  |  |  | .41 |
| Dorsolateral Prefrontal Cortex | 9 | R |  |  |  |  |  |  | .38 |
| Pre-motor and Supplementary | 6 | R |  |  |  |  |  |  | .21 |
| Pre-motor and Supplementary |  | L | -10 | -32 | 76 | 308 | -2.44 | .0118 |  |
| Pre-motor and Supplementary | 6 | L |  |  |  |  |  |  | .43 |
| Primary Motor Cortex | 4 | L |  |  |  |  |  |  | .30 |
| Primary Somatosensory Cortex | 3 | L |  |  |  |  |  |  | .20 |
| Superior Temporal Gyrus |  | R | 68 | -46 | 6 | 211 | -3.11 | .0027 |  |
| Superior Temporal Gyrus | 22 | R |  |  |  |  |  |  | .57 |
| Middle Temporal Gyrus | 21 | R |  |  |  |  |  |  | .39 |
| Superior Temporal Gyrus |  | L | -64 | -56 | 14 | 169 | -2.34 | .0147 |  |
| Superior Temporal Gyrus | 22 | L |  |  |  |  |  |  | .42 |
| Middle Temporal Gyrus | 21 | L |  |  |  |  |  |  | .23 |
| Angular Gyrus | 39 | L |  |  |  |  |  |  | .17 |
| Supramarginal Gyrus | 40 | L |  |  |  |  |  |  | .11 |
| Supramarginal Gyrus |  | R | 64 | -38 | 40 | 136 | 2.69 | .0068 |  |
| Supramarginal Gyrus | 40 | R |  |  |  |  |  |  | .88 |
| Visual Association Cortex (V2) |  | L | -30 | -86 | .28 | 133 | 2.34 | .0146 |  |
| Visual Association Cortex (V2) | 18 | L |  |  |  |  |  |  | 1.0 |
| Pre-motor and Supplementary |  | L | -22 | 22 | 64 | 86 | 3.50 | .0010 |  |
| Pre-motor and Supplementary | 6 | L |  |  |  |  |  |  | .76 |
| Frontal Eye Fields | 8 | L |  |  |  |  |  |  | .24 |

Supplementary Table 7. Whole-brain results for stuttered > fluent analysis (anticipation phase) (stutterers only) (oxyhemoglobin [HbO]). Regions on first line indicate locations of peak within cluster; indented lines represent other regions within cluster (Prob = probability, or the relative contributions of each region within the cluster). Positive t-values indicate anticipated > unanticipated; negative t-values indicate anticipated < unanticipated. Hemi = hemisphere; MNI = Montreal Neurological Institute.

| Region | Brodmann Area | Hemi | Peak MNI | | | Voxels | t | p | Prob |
| --- | --- | --- | --- | --- | --- | --- | --- | --- | --- |
|  |  |  | x | y | z |  |  |  |  |
| Somatosensory Association Cortex |  | R | 28 | -42 | 70 | 4,310 | 4.67 | .0001 |  |
| Somatosensory Association Cortex | 5 | R |  |  |  |  |  |  | .33 |
| Primary Somatosensory Cortex | 3 | R |  |  |  |  |  |  | .23 |
| Somatosensory Association Cortex | 7 | R |  |  |  |  |  |  | .17 |
| Primary Somatosensory Cortex | 2 | R |  |  |  |  |  |  | .14 |
| Pre-motor and Supplementary |  | R | 66 | -16 | 38 | 965 | -2.87 | .0046 |  |
| Pre-motor and Supplementary | 6 | R |  |  |  |  |  |  | .40 |
| Primary Somatosensory cortex | 1 | R |  |  |  |  |  |  | .17 |
| Primary Somatosensory Cortex | 2 | R |  |  |  |  |  |  | .14 |
| Primary Somatosensory Cortex | 3 | R |  |  |  |  |  |  | .14 |
| Middle Temporal gyrus |  | L | -60 | -18 | -14 | 573 | 2.51 | .0103 |  |
| Middle Temporal gyrus | 21 | L |  |  |  |  |  |  | .81 |
| Inferior Temporal gyrus | 20 | L |  |  |  |  |  |  | .19 |
| Frontal Eye Fields |  | L | -8 | 44 | 54 | 341 | -2.45 | .0116 |  |
| Frontal Eye Fields | 8 | L |  |  |  |  |  |  | .90 |
| Pre-motor and Supplementary |  | L | -40 | 4 | 62 | 304 | -3.42 | .0013 |  |
| Pre-motor and Supplementary | 6 | L |  |  |  |  |  |  | .95 |
| Dorsolateral Prefrontal Cortex |  | L | -26 | 36 | 34 | 193 | -2.26 | .0174 |  |
| Dorsolateral Prefrontal Cortex |  | L |  |  |  |  |  |  | .66 |
| Frontal Eye Fields |  | L |  |  |  |  |  |  | .34 |
| Frontopolar area |  | R | 26 | 64 | 42 | 161 | -2.46 | .0113 |  |
| Frontopolar area | 10 | R |  |  |  |  |  |  | 1.0 |
| Visual Association Cortex (V2) |  | R | 8 | -102 | -6 | 154 | -2.93 | .0040 |  |
| Visual Association Cortex (V2) | 18 | R |  |  |  |  |  |  | .83 |
| Primary Visual Cortex (V1) | 17 | R |  |  |  |  |  |  | .17 |
| Frontopolar area |  | R | 34 | 58 | 10 | 153 | -2.12 | .0229 |  |
| Frontopolar area | 10 | R |  |  |  |  |  |  | .98 |
| Dorsolateral Prefrontal Cortex |  | R | 46 | 36 | 22 | 63 | -1.96 | .0316 |  |
| Dorsolateral Prefrontal Cortex | 46 | R |  |  |  |  |  |  | .84 |
| Dorsolateral Prefrontal Cortex |  | L | -54 | 15 | 40 | 57 | -3.39 | .0014 |  |
| Dorsolateral Prefrontal Cortex | 9 | L |  |  |  |  |  |  | .52 |
| Pre-motor and Supplementary | 6 | L |  |  |  |  |  |  | .24 |
| Frontal Eye Fields | 8 | L |  |  |  |  |  |  | .22 |
| Frontal Eye Fields |  | R | 32 | 34 | 52 | 51 | 2.51 | .0102 |  |
| Frontal Eye Fields | 8 | R |  |  |  |  |  |  | .89 |
